# Phenotypic and Genomic Characterization of a Sulfate-Reducing Bacterium *Pseudodesulfovibrio methanolicus* sp. nov. Isolated from a Petroleum Reservoir in Russia

**DOI:** 10.3390/biology13100800

**Published:** 2024-10-07

**Authors:** Salimat K. Bidzhieva, Tatyana P. Tourova, Vitaly V. Kadnikov, Salima R. Samigullina, Diyana S. Sokolova, Andrey B. Poltaraus, Alexander N. Avtukh, Vera M. Tereshina, Alexey V. Beletsky, Andrey V. Mardanov, Tamara N. Nazina

**Affiliations:** 1 Winogradsky Institute of Microbiology, Research Center of Biotechnology, Russian Academy of Sciences, Moscow 119071, Russia; salima.bidjieva@gmail.com (S.K.B.); tptour@rambler.ru (T.P.T.); samigullinasalimar@gmail.com (S.R.S.); sokolovadiyana@gmail.com (D.S.S.); v.m.tereshina@inbox.ru (V.M.T.); 2 Institute of Bioengineering, Research Center of Biotechnology, Russian Academy of Sciences, Moscow 119071, Russia; vkadnikov@bk.ru (V.V.K.); mortu@yandex.ru (A.V.B.); mardanov@biengi.ac.ru (A.V.M.); 3 Engelhardt Institute of Molecular Biology, Russian Academy of Sciences, Moscow 119991, Russia; abpolt@gmail.com; 4 Skryabin Institute of Biochemistry and Physiology of Microorganisms, Russian Academy of Sciences, Pushchino Scientific Center for Biological Research of the Russian Academy of Sciences, Pushchino 142290, Moscow Region, Russia; avtukh@rambler.ru

**Keywords:** *Pseudodesulfovibrio*, sulfate-reducing bacteria, genomics, petroleum reservoir

## Abstract

**Simple Summary:**

Corrosion of steel equipment during oil production, transportation, and refining is a big global issue, leading to significant economic losses. The main agents of microbially influenced corrosion (MIC) of steel equipment are sulfate-reducing bacteria (SRB) and archaea (SRA), which reduce sulfate present in the reservoir water to form sulfide. Timely detection of sulfidogens in formations is necessary for the development of measures to suppress their growth. Existing 16S rRNA gene-based molecular methods for the detection of sulfidogens make it possible to identify them in their natural habitat, and the methods based on metagenomic analysis of components of the microbial community make it possible to predict their potential functional activity. However, selection of biocides or other methods for suppressing the growth of sulfidogens requires confirmation of their effectiveness on enrichment and/or pure cultures. In order to establish a collection of sulfidogens inhabiting the oil reservoirs of Tatarstan (Russia), a number of strains of sulfate-reducing bacteria were isolated. This study describes the 5S69^T^ strain, which, based on the physiological and biochemical characteristics and genomic analysis, has been assigned to a new species, *Pseudodesulfovibrio methanolicus* sp. nov. The strain is able to grow at high salinity, at reservoir temperature, and on media with alcohols or H_2_/CO_2_ in the presence of acetate, which indicates its adaptation to environmental conditions and potential in sulfide production in the oil reservoir.

**Abstract:**

The search for the microorganisms responsible for sulfide formation and corrosion of steel equipment in the oil fields of Tatarstan (Russia) resulted in the isolation of a new halotolerant strictly anaerobic sulfate-reducing bacterium, strain 5S69^T^. The cells were motile curved Gram-negative rods. Optimal growth was observed in the presence of 2.0–4.0% (*w*/*v*) NaCl, at pH 6.5, and at 23–28 °C under sulfate-reducing conditions. The isolate was capable of chemoorganotrophic growth with sulfate and other sulfoxides as electron acceptors, resulting in sulfide formation; and of pyruvate fermentation resulting in formation of H_2_ and acetate. The strain utilized lactate, pyruvate, ethanol, methanol, fumarate, and fructose, as well as H_2_/CO_2_/acetate for sulfate reduction. The genome size of the type strain 5S69^T^ was 4.16 Mb with a G + C content of 63.0 mol%. On the basis of unique physiological properties and results of the 16S rRNA gene-based phylogenetic analysis, phylogenomic analysis of the 120 conserved single copy proteins and genomic indexes (ANI, AAI, and dDDH), assigning the type strain 5S69^T^ ((VKM B-3653^T^ = KCTC 25499^T^) to a new species within the genus *Pseudodesulfovibrio*, is suggested, with the proposed name *Pseudodesulfovibrio methanolicus* sp. nov. Genome analysis of the new isolate showed several genes involved in sulfate reduction and its sulfide-producing potential in oil fields with high saline formation water.

## 1. Introduction

Sulfate-reducing bacteria and archaea are important components of the anaerobic branch of the sulfur cycle in freshwater, marine, and subsurface ecosystems [1,2]. The discovery of sulfate-reducing bacteria (SRB) in oil reservoirs about a hundred years ago by Bastin E.S. [3] and Ginzburg-Karagicheva T.L [4] gave rise to petroleum microbiology as a science. The exploitation of oil reservoirs using water-flooding leads to the activation of microbial processes in the reservoir, particularly those associated with reduction of sulfates [5,6,7,8]. This leads to emergence of sulfide in reservoir water, oil, and gas, deterioration of the environmental situation, and an increase in the cost of oil refining [9,10,11]. Due to intensive oil production around the world, interest in sulfate-reducing bacteria and archaea is increasing [12,13,14,15]. In this regard, it is necessary to control the composition of the microbial community in oil reservoirs.

This study is a part of the work on the isolation of sulfidogens from the oil reservoirs of Tatarstan (Russia) in order to find the ways to suppress their growth and minimize corrosion of oilfield equipment. Microorganisms of the Tatarstan oil reservoirs have been the object of research for more than 60 years. They have been studied using cultural, radioisotope, and molecular methods [16,17,18,19,20]. For example, in the water samples from Romashkinskoe and Vostochno-Anzirskoe petroleum reservoirs, the number of cultivated SRB reached 10^6^ and 10^4^ cells·mL^−1^; the sulfate-reduction rate estimated by radiotracer methods was 2.3–26.6 and 0.065–0.198 µg S^2–^·L^−1^ day^−1^, respectively [21,22]. Cloning of the 16S rRNA genes in the carbonate low-temperature bed 302 of the Romashkinskoe oil field revealed sulfate reducers of the genera *Desulfoglaeba*, *Desulfomicrobium*, *Desulfovibrio*, and unclassified members of the order *Desulfobacterales* [21]. By metabarcoding the V3–V4 region of the 16S rRNA gene sequences, sulfate-reducing bacteria of the genera *Desulfocurvus*, *Desulfotignum*, *Desulfonatronovibrio*, *Desulfovibrio*, *Desulfosalsimonas*, *Desulfovermiculus*, and *Desulfoglaeba* were detected in formation water from the Arkhangelskoe petroleum reservoir [22]. Using metagenomic analysis, metagenome-assembled genomes (MAGs) of SRB of the phylum *Desulfobacterota* assigned to the families *Desulfoplanaceae* (genus *Desulfoplanes*), *Desulfobacteraceae* (genus *Desulfotignum*), and *Desulfovibrionaceae* (genus *Pseudodesulfovibrio*) were reconstructed in reservoir water samples from the bed 302 of the Romashkinskoe oilfield [23].

A sulfate-reducing bacterial strain 5S69^T^ was isolated from a water sample obtained at the Vostochno-Anzirskoe oil field (Tatarstan, Russia). The strain had 93.0–99.5% 16S rRNA gene sequence similarity with the respective genes of bacteria of the genus *Pseudodesulfovibrio* and was preliminary assigned to this genus.

The genus *Pseudodesulfovibrio* was proposed by Cao and co-authors in 2016 for description of a mesophilic and piezophilic sulfate-reducing strain as a new species *Pseudodesulfovibrio indicus* [24]. The strain was isolated from a serpentinized peridotite sample from the Indian Ocean. Based on 16S rRNA gene sequence analysis of the paraphyletic genus *Desulfovibrio*, four species have been transferred to the genus *Pseudodesulfovibrio*—*Pseudodesulfovibrio piezophilus*, *Pseudodesulfovibrio profundus*, *Pseudodesulfovibrio portus*, and *Pseudodesulfovibrio aespoeensis* [24]. Although the species “*Pseudodesulfovibrio dechloracetivorans*”, which was not validly published [25], is also included in the *Pseudodesulfovibrio* cluster, the strain is currently missing from collections. A range of new SRB isolated from various environments was later described as members of this genus—*Pseudodesulfovibrio hydrargyri* [26], *Pseudodesulfovibrio halophilus* [27,28], *Pseudodesulfovibrio mercurii* [29], *Pseudodesulfovibrio tunisiensis* [30], “*Pseudodesulfovibrio cashew*” [31], “*Pseudodesulfovibrio thermohalotolerans*” [32], “*Pseudodesulfovibrio pelocollis*” [33], and others. At the time of writing, the genus *Pseudodesulfovibrio* comprised 13 species with a validly published and correct name and five species not validly published under the International Code of Nomenclature of Bacteria (ICNP) https://lpsn.dsmz.de/genus/pseudodesulfovibrio (accessed on 1 August 2024) [34,35]. The genus *Pseudodesulfovibrio* belongs to the family *Desulfovibrionaceae*, order *Desulfovibrionales*, class *Deltaproteobacteria* of the phylum *Pseudomonadota* [24,36].

Phenotypically, members of the genus *Pseudodesulfovibrio* are very similar. They are represented by anaerobic, Gram-negative, non-spore-forming vibrios or curved rods reducing sulfate, thiosulfate, and sulfite to hydrogen sulfide and using a small range of organic compounds or molecular hydrogen + CO_2_ as donors of electrons and carbon. Members of this genus were isolated from marine and brackish lake sediments, terrestrial mud volcano, oil refinery water, and production water of oil fields [24,32]. The G + C content of the chromosomal DNA varies in a wide range of 50–63.5 mol% [24], showing that the genus *Pseudodesulfovibrio* appears to be paraphyletic. As a result of the ongoing revision of the genus *Pseudodesulfovibrio*, the species *P. halophilus* and *P. senegalensis* were transferred to a new genus *Salidesulfovibrio* as “*Salidesulfovibrio halophilus*” and “*Salidesulfovibrio senegalensis*” [37].

The purpose of this work was characterization of morphology, physiology, and chemotaxonomic features of the strain 5S69^T^ isolated from a petroleum reservoir (Tatarstan, Russia), as well as genomic analysis to determine its taxonomic affiliation. In this study, we present new information on phenotype of the sulfate-reducing strain 5S69^T^ supplemented by genome sequencing and phylogenomic analysis. Estimation of the overall genomic relatedness indices (OGRI) as primary molecular criteria strongly supported the conclusion on affiliation of the strain 5S69^T^ to a new species of the genus *Pseudodesulfovibrio*. Thus, *Pseudodesulfovibrio methanolicus* sp. nov. was proposed with type strain 5S69^T^ (=VKM B-3653^T^ = KCTC 25499^T^). The genome analysis of strain 5S69^T^ confirmed the phenomenological observations and revealed the genes determining the strain’s adaptation to the conditions of the oil reservoir.

## 2. Materials and Methods

### 2.1. Strain Isolation and Cultivation

Strain 5S69^T^ (=VKM B-3653^T^ = KCTC 25499^T^ = UQM 41509^T^) was isolated from an injection water sample obtained in June 2016 at Vostochno-Anzirskoe oil field (55°66′69″ N, 51°49′84.00″ E), located in Yelabuga district, Tatarstan (Russia). The oilfield is exploited with water-flooding using a mixture of fresh river water and formation water separated from oil [38]. Formation water of the chlorine-calcium type is characterized by a high total salinity around 100 g·L^−1^. The oil stratum was represented by a terrigenous sandstone oil-bearing Devonian deposit located at the depth of about 1585 m below sea level and having a temperature about 23 °C. In the reservoir water samples from production wells, the number of sulfate-reducing bacteria reached 10^4^ cells/mL and the rate of sulfate reduction varied from 65 to 1982 ng sulfide L^−1^·day^−1^ [22,38]. According to analyses of surface samples, oil density was 0.856 g·cm^−3^ (at 20 °C). Anaerobic sulfate-reducing enrichment culture was obtained in Postgate’s B medium with sodium lactate (3.5 g·L^−1^), supplemented with microelements and reduced with Na_2_S·9H_2_O (200 mg·L^−1^) as described previously [22]. The pure culture was isolated by repeated tenfold dilutions in a liquid marine Widdels’ medium [39] at 25 °C by dilution to extinction method until obtaining axenic isolates. The purity of the cultures was checked by phase-contrast microscopy of wet biomass and by analysis of the 16S rRNA gene sequence of the culture. The purity of type strain was checked in two culture collections also (=VKM B-3653^T^ = KCTC 25499^T^).

### 2.2. Morphological, Physiological, and Chemotaxonomic Characterization

To determine the morphological, physiological, and chemotaxonomic properties, strain 5S69^T^ was cultured in anaerobic conditions on a mineral medium (MM) containing (per 1 L distilled water): 0.2 g KH_2_PO_4_, 0.25 g NH_4_Cl, 20 g NaCl, 3.0 g MgCl_2_ · 6H_2_O, 0.5 g KCl, 1.5 g CaCl_2_ · 2H_2_O, 2.0 g Na_2_SO_4_, 0.5 g cysteine-HCl; pH 6.5. The medium was supplemented with 0.3 g·L^−1^ of yeast extract and 1 mL·L^−1^ each 0.1% (*w*/*v*) Mohr’s salt [FeSO_4_ · (NH_4_)_2_SO_4_ · 6H_2_O], solutions of vitamins [40] and microelements [41], and 3.5 g·L^−1^ sodium lactate as a carbon and electron source. The media were prepared using chemicals from Merck KGaA (Darmstadt, Germany). The medium was prepared anaerobically under a stream of Ar, dispensed into Hungate tubes [42], sealed with butyl rubber stoppers, and autoclaved at 121 °C for 60 min. To determine the temperature range for growth, the strain was cultivated at 5, 10, 15, 23, 28, 33, 37, and 42 °C. The effect of salinity was determined at NaCl concentrations of 0, 2, 5, 10, 20, 40, 60, 70, 80, 90, and 100 g·L^−1^. The growth of the strain was also studied in the pH range from 3.4 to 9.1. In each tube, the pH of the medium was adjusted to the desired value with sterile solutions of 1% HCl and 10% NaHCO_3_. Additional soluble components (substrates, electron acceptors, etc.) were added into the medium from sterile stock solutions. Substrate utilization tests were performed in MM medium with sulfate supplemented with 0.1 g·L^−1^ of yeast extract and 2 g·L^−1^ organic substrate (short-chain fatty acids, methanol, ethanol, pyruvate, fumarate, sugars, or amino acids). Thiosulfate, sulfur, fumarate (2 g·L^−1^ each) and nitrate (0.85 g·L^−1^) were tested as electron acceptors in MM medium (without sulfate) with 3.5 g·L^−1^ sodium lactate. Insoluble components (such as elemental sulfur) were added directly into test tubes during the preparation of a liquid nutrient medium. All of the incubations were carried out in anaerobic static conditions at 28 °C for 14 days except for the experiments on the determination of the temperature range for growth. Growth on H_2_/CO_2_ (4:1, vol./vol.) was estimated both with and without sodium acetate (0.1 g·L^−1^).

The growth of the strain was additionally tested under aerobic and microaerophilic conditions on MM medium with sodium lactate, which does not contain cysteine-HCl. The gas phase was air in different proportions with argon (5, 10, and 20% O_2_). All experiments were carried out in 3 repetitions.

The growth of the strain was controlled by turbidity of the medium on an Ultrospec 2100 pro spectrophotometer (Amersham Biosciences, Slough, UK) at a wavelength of 660 nm and by the increase in the concentration of sulfide, which was determined using the colorimetrical method by Trüper and Schlegel [43]. Fermentation products were determined using chromatographic separation techniques. Volatile fatty acids and alcohols were analyzed with a Shimadzu GC 2010 Plus gas chromatograph (Shimadzu, Kyoto, Japan) in a column (30 m × 0.32 mm) with a Zebron ZB-FFAP (Phenomenex Ltd., Aschaffenburg, Germany) phase thickness of 0.25 μm, as described previously [44]. Gaseous products of metabolism, H_2_ and CO_2_, were determined by gas chromatography. Nitrite was determined using the Griess reagent.

Cell morphology, growth, and physiological state of the cultures were assessed also under an Axio 105 Imager.D1 epifluorescence microscope (Carl Zeiss, Oberkochen, Germany). The scanning microscopy images of the cells were obtained using a JSM-IT200 scanning electron microscope (JEOL, Tokyo, Japan) (accelerating voltage 20 kV, High Vac) as described earlier [15]. Ultrathin sections of the strain were examined using a model JEM-100C transmission electron microscope (JEOL, Tokyo, Japan) at 80 kV as described previously [44].

For DNA isolation and analyses of isoprenoid quinones, cellular fatty acids, and polar lipids of the strain 5S69^T^, the biomass was obtained in MM medium with lactate and sulfate after 14 days of cultivation at 28 °C. The type strain *Desulfovibrio desulfuricans* subsp. *desulfuricans* Adams Essex 6 (=VKM B-1799^T^), obtained from the All-Russian Collection of Microorganisms (VKM; Pushchino, Moscow Region, Russia) was used for comparison. The strain VKM B-1799^T^ was cultured on the same medium as the 5S69^T^ strain, but containing 1.0 g L^−1^ NaCl. The cells of both strains were collected in the late exponential growth phase by centrifugation for 20 min at 3400× *g* and freeze-dried. The fatty acid composition was analyzed using a Maestro gas chromatograph-mass spectrometer (Interlab, Russia) as was described by Bidzhieva et al. [44]. Isoprenoid quinones were analyzed at the All-Russian Collection of Microorganisms as was described previously [45]. Quinones were extracted from wet cells according to Collins and Jones [46] and analyzed with a Thermo Finnigan LCQ Advantage MAX mass spectrometer (Thermo Fisher Scientific Inc., Waltham, MA, USA). The extract of polar lipids was obtained according to the method by Minnikin et al. [47]. Polar lipids were separated by two-dimensional thin-layer chromatography on silica gel layers [48]. Aminolipids, glycolipids, phospholipids, and choline containing lipids were identified using spraying with ninhydrin, α-naphthol, Dittmer–Lester molybdenum blue reagent (Vaskovsky modification), and Dragendorff reagent, respectively, as described previously [49].

### 2.3. 16S rRNA Gene and Genome Sequencing and Annotation

The Power Soil kit (MO BIO Laboratories, Carlsbad, CA, USA) was used to isolate genomic DNA of the strain 5S69^T^. Primers 27F and 1492R [50] were used to amplify the 16S rRNA gene of the strain 5S69^T^. Purified PCR products were sequenced on an ABI Prism 3730 DNA analyzer (Applied Biosystems, Foster City, CA, USA) using a Big Dye Terminator reagent kit version 3.1. The complete genome of the strain 5S69^T^ was obtained using a combination of Illumina MiSeq and MinIon (Oxford Nanopore Technologies, Oxford, UK)) single-molecule sequencing technologies. To prepare the shotgun genome library, the NEBNext Ultra II DNA Library preparation kit (New England BioLabs, Ipswich, MA, USA) was used. Sequencing of the NEBNext Ultra II DNA library generated 4,253,422 paired end reads (2 × 300 nt), for a total of 1,153,741,282 bases. Primers for sequencing were removed using Cutadapt v.1.8.3 [51], and low-quality read areas (mean q < 30) were cut using Sickle v.1.33 (https://github.com/najoshi/sickle, accessed on 24 July 2024) [52]. In addition, 432,002 nanopore reads with a total length of 1051,214,891 nt were obtained using the MinIon sequencer. The reads nanopore were de novo assembled into a complete ring chromosome using Flye v2.8 [53]. The consistent genome sequence was polished using two iterations of Pilon v.1.22 [54] with the reads mapping. Gene search and annotation were performed using the RAST 2.0 [55] server.

### 2.4. Bioinformatic Analysis

The 16S rRNA gene sequences were aligned using MOTHUR v.1.47.0 against the Silva v138 reference seed database. The 16S rRNA gene-based maximum-likelihood phylogenetic tree was constructed with FastTree v2.1.11 using the default Jukes-Cantor + CAT model and 20 rate categories of sites. Branch support values were calculated using the Shimodaira-Hasegawa test. Phylogenomic analysis was performed using concatenated sequences of 120 marker genes obtained and aligned using GTDB-Tk v2.3.2 [56]. Maximum likelihood phylogeny was estimated by PhyML v3.3 using the LG substitution model, 4 substitution rate categories described by gamma distribution with estimated shape parameter, branch support values were calculated by approximate Bayes method. The pangenomic analysis was performed based on a bioinformatic pipeline of Anvi’o version 8.0 [57]. Genomes were arranged accordingly to the maximum likelihood genome tree. The average nucleotide identity (ANI) between genomes of the strain 5S69^T^ and *Pseudodesulfovibrio* spp. was calculated using the ani.rb script from the Enveomics Collection [58]. Digital DNA-DNA hybridization (dDDH) of genomes was performed using the Genome-to-Genome Distance Calculator (GGDC) v. 2.1 online tool [59].

The possible metabolic pathways in *Pseudodesulfovibrio* spp. genomes were reconstructed using BV-BRC (PATRIC) 3.37.14 (https://www.bv-brc.org/, accessed on 1 July 2024) [60], MetaCyc version 28.0 (https://metacyc.org/, accessed on 2 April 2024) [61], RAST v. 2.0 (https://rast.nmpdr.org/rast.cgi, accessed on 12 October 2023) [62], and BlastKOALA annotation tool at the KEGG web resource version 3.0 (https://www.kegg.jp/blastkoala/, accessed on 18 April 2024) [63]. The selected gene clusters were compared using an online service Gene Graphics version 2.02 (https://katlabs.cc/genegraphics/app, accessed on 9 March 2023) [64]. The Proksee web service version 6.0.3 (https://proksee.ca/, accessed on 5 March 2024) was used for the construction of the genome map [65].

### 2.5. Nucleotide Sequence Accession Numbers

The GenBank accession number for the 16S rRNA gene sequence of strain 5S69^T^ is PP792559. The complete genome sequence of strain 5S69^T^ has been deposited at GenBank under the accession number CP146609.

## 3. Results and Discussion

In this study, we characterized the physiological and biochemical features of sulfate-reducing bacterial strain 5S69^T^, isolated from reservoir water of the Vostochno-Anzirskoe oil field (Russia). The strain was shown to be well-adapted to the temperature and salinity of its habitat. Genomic analysis revealed the potential metabolic functions of strain 5S69^T^, which were not detected during phenotypic studies, and to determine its taxonomic affiliation as a new species *Pseudodesulfovibrio methanolicus* sp. nov.

### 3.1. Phenotypic Characteristics of Strain 5S69^T^

**Figure 1:**
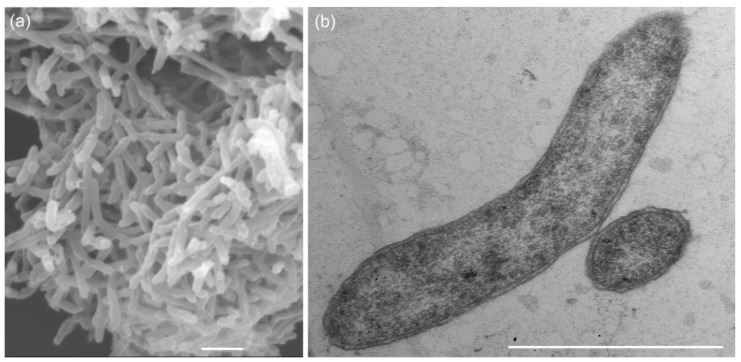
Scanning electron micrograph of total cells (**a**) and transmission electron micrograph of an ultrathin section (**b**) of the strain 5S69^T^ showing Gram-negative structure of the cells, containing an external lipoprotein membrane. The strain was grown in the medium with lactate and sulfate for 7 days at 25 °C. Bars, 1 µm.

Similar to members of the genus *Pseudodesulfovibrio*, strain 5S69^T^ was chemoorganotrophic, strictly anaerobic, and Gram-stain-negative. This strain could not use acetate as an energy and a carbon source and produced acetate on media with sulfate and organic substrates. In an exponential growth phase, cells of the strain were straight or slightly curved rods, 0.35–0.45 × 1.0–2.0 μm, motile by means of a single polar flagellum, non-spore-forming. Ultrathin sections showed the inner cytoplasmic and outer lipoprotein membranes, confirming the cell wall structure typical of Gram-negative bacteria (Figure 1). Optimal growth of strain 5S69^T^ was observed at 23–28 °C, 2% (*w*/*v*) NaCl, and at pH 6.5, i.e., under the conditions identical to those of the petroleum reservoir from which the strain was isolated (Figure S1). Growth occurred in a range of 15–37 °C, 0.2–6% (*w*/*v*) NaCl, and at pH 4.6–8.6. At temperatures below 15 °C and above 42 °C, growth was absent during cultivation for 22 days. The strain did not grow in media containing less than 2 g and more than 60 g of NaCl/L. The detailed characteristics of the strain 5S69^T^ and phylogenetically most closely related *Pseudodesulfovibrio* species are shown in Table 1.

Strain 5S69^T^ used sulfate, sulfite, thiosulfate, and fumarate, but not nitrate as electron acceptors. In the presence of molecular oxygen in the gas phase, no complete inhibition of growth was observed. The cells remained viable, but exhibited poor growth compared to growth under strictly anaerobic conditions. The optical density of a culture growing for 14 days in a reduced anaerobic nutrient medium in the presence of sulfate was 2–3 times higher than the optical density of a culture growing in aerobic and microaerophilic conditions. The strain carried out incomplete oxidation of organic substrates to acetate. Strain 5S69^T^ reduced sulfate to sulfide in media with lactate, pyruvate, formate, fumarate, malate, succinate, ethanol, methanol, glycerol, fructose, yeast extract, and peptone. Weak growth was also observed on glutamate, citrate, propanol, galactose, and mannose; however, the strain did not use acetate, propionate, butyrate, glycine, serine, ornithine, glucose, lactose, sucrose, or benzoate. The strain reduced sulfate in a medium with molecular hydrogen as an electron donor and CO_2_ and acetate as carbon sources for assimilation processes. In the absence of sulfate and other electron acceptors, strain 5S69^T^ fermented pyruvate, forming acetate, CO_2_, and molecular hydrogen; lactate was not fermented. No vitamins or other growth factors were required.

Cellular fatty acid compositions of strain 5S69^T^ and of the type strain *Desulfovibrio desulfuricans* B-1799^T^ (=DSM 642) are given in Table S1. Predominant acids in the fatty acid profile of the strain 5S69^T^ were *iso*-C_15:0_ (20.4%), *anteiso*-C_15:0_ (19.3%), and C_16:0_ (16.3%); in smaller quantities were detected C_17:1_ ω9t (6.6%), *iso*-C_16:0_ (4.2%), C_17:1_ ω9c (3.8%), C_18:1_ ω10 (3.7%), *iso*-C_17:0_ (3.5%), and *iso*-C_14:0_ (3.2%). *D. desulfuricans* VKM B-1799^T^ contained a large amount of *iso*-C_15:0_ (43.3%), C_17:1_ ω9c (34.8%), *iso*-C_17:0_ (8.6%), and C_16:0_ (7.1%). The major polar lipids of strain 5S69^T^ were phosphatidylethanolamines (PE), diphosphatidylglycerols (DPG), phosphatidylglycerols (PG), glycolipids (GL), and phosphatidylserines (PS) (Figures S2 and S3). In the polar lipids profile of strain *D. desulfuricans* VKM B-1799^T^ were detected PE, DPG, PG, GL, and PL (Figures S2 and S3). The major respiratory quinone in strain 5S69^T^ was menaquinone MK-6(H_4_).

### 3.2. Phylogenetic Analyses of the 16S rRNA Gene Sequences

**Figure 2:**
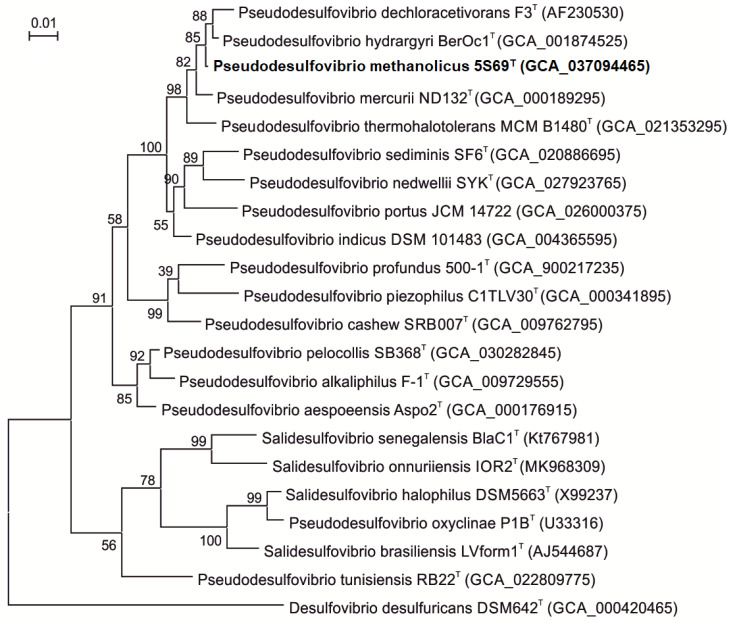
Phylogenetic tree based on the 16S rRNA gene sequences showing the taxonomic position of strain 5S69^T^ within the genus *Pseudodesulfovibrio*. The scale bar indicates 1 nt substitution per 100 nucleotides. The tree was rooted using *Desulfovibrio desulfuricans* strain DSM642^T^ as an outgroup. GenBank accession numbers for 16S rRNA genes are indicated in brackets. The name of the strain described in this study is marked by boldface.

Comparative analysis of the 16S rRNA gene sequence of strain 5S69^T^ (1386 bp, accession no. PP792559.1) obtained by Sanger sequencing showed the highest sequence similarities with genes of the type strains ‘*Pseudodesulfovibrio dechloracetivorans*’ SF3^T^ (98.7%), *Pseudodesulfovibrio hydrargyri* BerOc1^T^ (99.5%), and ‘*Pseudodesulfovibrio thermohalotolerans’* MCM B1480^T^ (98.4%). Strain 5S69^T^ was distantly related to the type strain of the type species of the genus *Desulfovibrio*, *Desulfovibrio desulfuricans* Essex 6 (89.4% similarity). On the 16S rRNA-based phylogenetic tree, constructed using complete 16S rRNA gene sequences from the genomes, strain 5S69^T^ formed a distinct cluster within the *Pseudodesulfovibrio* clade (Figure 2). The trees topology evaluated by using the maximum-likelihood and the neighbor-joining methods was almost the same, supporting affiliation of strain 5S69^T^ to the genus *Pseudodesulfovibrio* as a separate species.

According to the trees constructed using the neighbor-joining and maximum likelihood algorithms, the genus *Pseudodesulfovibrio* appears to be polyphyletic. The tree topology supports transfer of the species *P. halophilus* and *P. senegalensis* to a new genus *Salidesulfovibrio* as “*Salidesulfovibrio halophilus*”, and “*Salidesulfovibrio senegalensis*” [37]. Further taxonomic revision of the genus *Pseudodesulfovibrio* may be required to resolve the evolutionary relationships between members of this group.

### 3.3. Genome Features and Phylogeny

To clarify the taxonomic status of the strain 5S69^T^, its genome was sequenced and analyzed. The complete genome sequence of strain 5S69^T^ (accession number GCF_037094465.1) was composed in one chromosome with a total length of 4,161,161 bp, a G + C content of 63%, and coverage of 314×. The genome completeness and contamination were assessed as 98.3% (100th percentile) and 2.41%, respectively. The genome comprises 3890 annotated genes, including 3803 protein-coding sequences, 19 pseudogenes, and 68 RNA genes. The genome contains two copies each of the 16S rRNA and 23S rRNA genes. The 16S rRNA gene sequence of strain 5S69^T^ obtained by PCR amplification (1386 bp, accession no. PP792559) and the sequences of respective regions of 16S rRNA genes obtained from the genome (1559 and 1559 bp) showed 100% identity, which confirmed the authenticity of the final genome assembly. The ANI and dDDH values of the 5S69^T^ genome and the genomes of three phylogenetically most closely related *Pseudodesulfovibrio* type strains, *P. hydrargyri* BerOc1^T^, *P. thermohalotolerans* MCM B-1480^T^, and *P. indicus* J2^T^, were in a range 83.7–89.9% and 26.2–40.3%, respectively (Table 1). These values were below the thresholds of 95–96% for ANI and 70% for dDDH, accepted for the delineation of bacterial species [66,67], which indicates that strain 5S69^T^ belongs to a new species.

**Figure 3:**
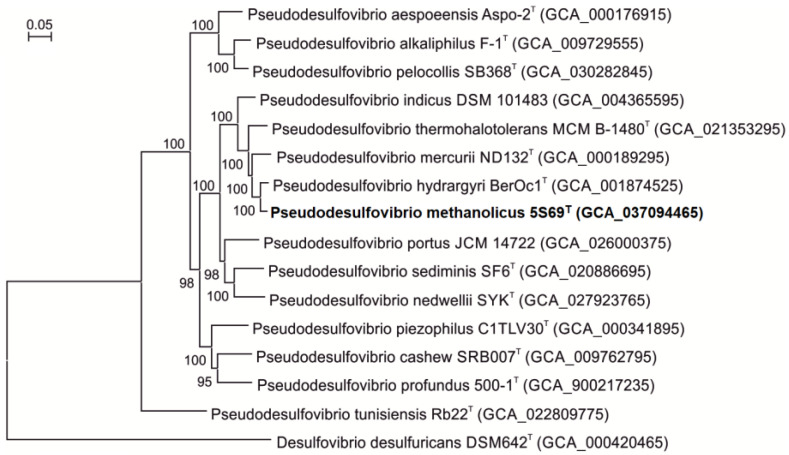
Phylogenomic placement of sulfate-reducing strain 5S69^T^ within the genus *Pseudodesulfovibrio* based on concatenated amino acid sequences of 120 single-copy proteins. The tree was reconstructed using the Maximum Likelihood algorithm. Bar: 0.05 amino acid substitutions per site. The tree was rooted using *Desulfovibrio desulfuricans* DSM642^T^ as an outgroup. Accession numbers for the genomic assemblies are indicated in brackets. The name of the strain described in this study is marked by boldface.

On the phylogenetic tree constructed based on concatenated 120 single-copy proteins strain 5S69^T^ formed an independent branch within the genus *Pseudodesulfovibrio*, indicating that the new strain could represent a new member of the genus *Pseudodesulfovibrio* (Figure 3).

**Figure 4:**
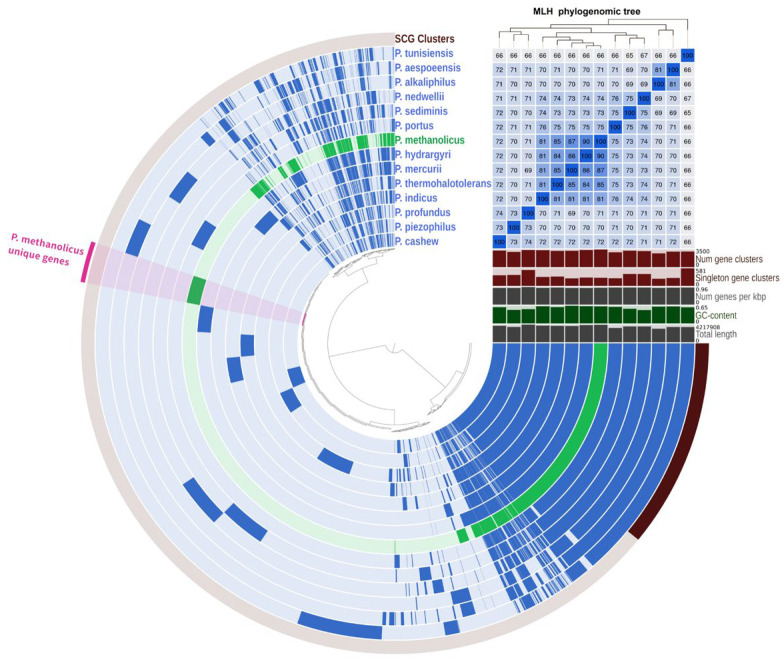
Pangenome analysis of 9515 gene clusters (49,203 genes) showing relationships between genomes of strain 5S69^T^ and members of the genus *Pseudodesulfovibrio*. Dark circular regions represent genes found in those areas for each genome. The phylogenetic tree is reconstructed using the single copy genes. AAI heatmap in blue squares varies between 65 and 100%.

Pangenomic analysis was performed on 14 genomes of bacteria of the genus *Pseudodesulfovibrio*. The dataset composed of 49,203 genes was organized into 9515 gene clusters using Anvio-8. The core genome was represented by 1664 gene clusters, of which 1381 were single-copy genes (Figure 4). The genome of strain 5S69^T^ possessed 273 unique genes not occurring in other *Pseudodesulfovibrio* species, 119 of which had a function predicted according to the KEGG database. These unique genes highlight the specialized adaptations of strain 5S69^T^, particularly in nutrient transport, lipopolysaccharide biosynthesis, nucleotide sugar biosynthesis, and defense mechanisms.

Among the unique genes, the one encoding citrate (pro-3S)-lyase (EC: 4.1.3.6) was detected (Table S2). Citrate lyase is an enzyme which converts citrate to oxaloacetate. In bacteria, this reaction is involved in citrate fermentation to acetate. This citrate lyase complex was most similar to that of members of the genus *Desulfovibrio*, which can indicate horizontal transfer. In addition to this complex, phosphoglycerate kinase and NAD(P)-dependent glyceraldehyde-3-phosphate dehydrogenase genes were also detected. This is one of the main differences between strain 5S69^T^ and other *Pseudodesulfovibrio* species, which enriches our understanding of its unique physiological capabilities and metabolic diversity within the genus.

### 3.4. Genome Analysis and Functional Annotation

**Figure 5:**
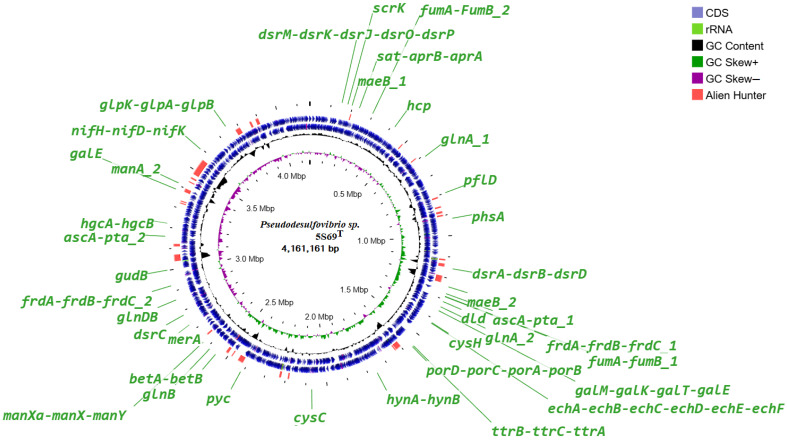
Circular genome map of the strain 5S69^T^. Abbreviations: *dsrMKJOP*, [DsrC]-trisulfide reductase; *scrK*, fructokinase; *maeB_1-2*, malate dehydrogenase; *sat*, sulfate adenylyltransferase; *aprAB*, adenylylsulfate reductase; *hcp*, hydroxylamine reductase; *glnA_1-2*, glutamine synthetase; *pflD*, pyruvate formate-lyase; *phsA*, thiosulfate reductase/polysulfide reductase; *dsrABD*, dissimilatory sulfite reductase; *askA_1-2*, acetate kinase; *pta_1-2*, phosphate acetyltransferase; *fumAB_1-2*, fumarate hydratase; *frdABC_1-2*, succinate dehydrogenase; *dld*, D-lactate dehydrogenase; *galM*, aldose 1-epimerase; *galK*, galactokinase; *galT*, UDPglucose–hexose-1-phosphate uridylyltransferase; *cysH*, phosphoadenosine phosphosulfate reductase; *echABCDEF*, multisubunit energy-conserving, membrane-bound Ech-[NiFe]-hydrogenase (ferrodoxin); *ttrBCA*, tetrathionate reductase; *porDCAB*, pyruvate ferredoxin oxidoreductase; *hynAB*, HynAB-type cytochrome-c3 [NiFe] hydrogenase; *cysC*, adenylyl-sulfate kinase; *pyc*, pyruvate carboxylase; *betA*, choline dehydrogenase; *betB*, betaine-aldehyde dehydrogenase; *merA*, mercuric ion reductase; *glnB*, *glnAB*, glutamate synthase; *manXaXY*, mannose PTS system; *dsrC*, sulfur redox associated protein; *gudB*, glutamate dehydrogenase; *galE*, UDP-glucose 4-epimerase; *hgcAB*, mercury methylation corrinoid and ferredoxin-like proteins; *nifH*, reductase nitrogenase; *nifDK*, nitrogenase; *glpK*, glycerol kinase*; glpAB*, anaerobic glycerol-3-phosphate dehydrogenase.

The genome analysis of strain 5S69^T^ revealed genes for all enzymes of the pathways of glycolysis (Embden-Meyerhoff pathway) and glucogenesis, pyruvate oxidation, pentose phosphate pathway, fructose, mannose, and galactose degradation, and UDP-glucose and UDP-galactose biosynthesis. Localization of the genes presumably involved in the major metabolic pathways is shown on the circular genome map (Figure 5).

The citrate cycle of strain 5S69^T^ is not closed; only the enzymes of first carbon oxidation are fully represented. A complete set of genes for enzyme complexes of ATP synthesis has also been identified: NADH-ubiquinone oxidoreductase (EC: 7.1.1.2), F-type ATPase (EC: 7.1.2.2) and cytochrome *c* oxidase. Other revealed genes were responsible for biosynthesis of basic amino acids; dissimilatory sulfate reduction; fixation of molecular nitrogen; biosynthesis and beta-oxidation of fatty acids; phosphate acetyltransferase-acetate kinase pathway; phosphatidylethanolamine (PE) biosynthesis; lipopolysaccharide biosynthesis: dTDP-L-rhamnose biosynthesis; terpenoid backbone biosynthesis; as well as the biosynthesis of vitamins and cofactors: thiamine salvage pathway, coenzyme A, FAD, and NAD. At the same time, genes encoding the enzymes of complete xenobiotic degradation pathways have not been identified.

According to the results of the built-in Alien Hunter module, a small number of sections of foreign DNA were revealed, presumably obtained via horizontal gene transfer. None of the genes of the metabolic pathways discussed in this paper are localized in these sites, which indicates their vertical evolution.

#### 3.4.1. Carbohydrate Metabolism and Oxidation of Organic Compounds

The ability of the strain 5S69^T^ to grow on fructose and mannose in the presence of sulfates according to the BlastCOALA service was confirmed by the presence of the fructose catabolism gene *scrK* (V8V93_00875), encoding fructokinase (EC: 2.7.1.4), as well as mannose catabolism genes *manXaXY* (V8V93_12505–12495), coding mannose PTS system (EC: 2.7.1.191), and *manA* (V8V93_09910, V8V93_16175), encoding mannose-6-phosphate isomerase (EC: 5.3.1.8), catalyzing the transformation of these sugars to D-fructose 6-phosphate, which is further catabolized via glycolysis (Figure S4). In addition, the genome of strain 5S69^T^ presumably contains a complete set of genes encoding the enzymes of galactose degradation, Leloir pathway (Figure S5), which includes the genes *galM* (V8V93_06295), encoding aldose 1-epimerase (EC: 5.1.3.3), *galK* (V8V93_06285), encoding galactokinase (EC: 2.7.1.6), *galT* (V8V93_06280), encoding UDPglucose--hexose-1-phosphate uridylyltransferase (EC: 2.7.7.12), and *galE* (V8V93_16075), encoding UDP-glucose 4-epimerase (EC: 5.1.3.2). The degradation products of galactose can subsequently be incorporated into glycolysis and other carbohydrate metabolism pathways. Strain 5S69^T^ was not able to grow on sucrose as an electron source in the presence of sulfates, which is consistent with the absence of the genes of the relevant enzymes in its genome.

Strain 5S69^T^ oxidized organic substrates such as lactate, pyruvate, malate, fumarate, and succinate during sulfate reduction. In the genome of the strain, the gene *dld* (V8V93_06090) was annotated, encoding predicted enzyme D-lactate dehydrogenase (Fe-S protein, FAD/FMN-containing dehydrogenase, EC:1.1.99.6), which catalyzes the oxidation reaction of D-lactate to pyruvate (Figure S6). Pyruvate ferredoxin oxidoreductase (EC: 1.2.7.1) is presumably used for pyruvate oxidation, which is encoded by cluster genes *porDCAB* (V8V93_07195–07205). Note that homologous clusters of genes of this enzyme are annotated only in the genomes of *P. indicus* J2^T^ and *P. hydrargyri* BerOc1^T^ phylogenetically close to strain 5S69^T^. In addition, duplicated genes *maeB* (V8V93_00960; V8V93_05795) are present in the genome of the strain 5S69^T^, as well as the genes *fumAB* (V8V93_05980–05985; V8V93_14000–14005) and *frdABC* (V8V93_05990–06000; V8V93_14010–14020), coding malate dehydrogenase (NADP+) (EC: 1.1.1.40), fumarate hydratase (EC: 4.2.1.2), and succinate dehydrogenase (EC: 1.3.5.1), respectively, which may be involved in oxidation of malate, fumarate, and succinate to pyruvate. The genome of strain 5S69^T^, similar to all other known species of the genus *Pseudodesulfovivrio*, lacks the gene *mdh*, encoding malate dehydrogenase (EC: 1.1.1.37), which catalyzes the interconversion of malate to oxaloacetate utilizing the NAD/NADH cofactor system. Presumably to replenish oxaloacetate in the tricarboxylic acid cycle, the pyruvate carboxylase enzyme (EC: 6.4.1.1) encoded by the *pyc* gene (V8V93_11430) is used in the metabolism of the strain 5S69^T^, as in other *Pseudodesulfovivrio*.

The inability of strain 5S69^T^ to use propionate and butyrate was a characteristic feature of the type strains of other *Pseudodesulfovibrio* species, including *P. indicus*, the type species of the genus [24], as was confirmed by the absence of the annotated genes for propanoyl- and butanoyl-CoA degradation in their genomes.

#### 3.4.2. Hydrogen Utilization and CO2 Fixation

Strain 5S69^T^ was not able to grow autotrophically on molecular hydrogen and carbon dioxide in the presence of sulfate. However, the growth on hydrogen was observed when acetate was added to the medium. This ability has been shown previously for *Desulfovibrio vulgaris* Marburg (=*Nitratidesulfovibrio vulgaris*) using radiotracer analysis [68]. At the same time, acetate and additionally CO_2_ were used in the process of growth only as carbon sources for biosynthesis of the cellular components. Subsequently, the ability to grow under similar conditions was shown for some heliobacteria [69]. Presumably, using pyruvate synthase (or pyruvate: ferredoxin oxidoreductase) (EC: 1.2.7.1), the reverse reaction of pyruvate oxidation is carried out, in which acetyl-CoA is converted to pyruvate with the inclusion of CO_2_ using reduced ferredoxin (Figure S7). The activation of acetate to acetyl-CoA can occur using the reversed phosphate acetyltransferase-acetate kinase pathway; the genes coding this enzyme are annotated in the genome of the strain 5S69^T^. The enzyme acetate kinase (EC: 2.7.2.1), encoded by the *ascA* gene, phosphorylates acetate to acetyl phosphate, and the enzyme phosphate acetyltransferase (EC: 2.3.1.8), encoded by the *pta* gene, transforms acetyl phosphate to acetyl-CoA. The *ascA-pta* gene cluster is present in the genome of strain 5S69^T^ in two copies (V8V93_05925–05930 and 15095–15100). According to the BlastCOALA service, the genes of enzymes providing this pathway of metabolism are annotated in the genomes of all type strains of the species of the genus *Pseudodesulfovibrio*.

#### 3.4.3. Glycerol and Alcohols Utilization

Strain 5S69^T^ was able to grow on glycerol using it as an electron donor for sulfate reduction. The genome of the strain 5S69^T^ contains the *glpK* gene (V8V93_17545), which encodes the glycerol kinase enzyme (EC: 2.7.1.30), which, according to the MetaCyc portal, performs the first stage of the glycerol degradation I pathway. At this stage, glycerol is transformed with the participation of ATP to sn-glycerol 3-phosphate. The *glpK* gene is part of a complete cluster of glycerol metabolism genes (V8V93_17545–17600), including also the *glpAB* genes coding the enzyme anaerobic glycerol-3-phosphate dehydrogenase (EC: 1.1.5.3), which catalyzes the second stage of the glycerol degradation I pathway to glycerone phosphate. The latter is subsequently catabolized via glycolysis and glucogenesis. In addition to the genes of these enzymes, the glycerol metabolism cluster includes the *glkR* gene encoding glycerol-3-phosphate regulon repressor gene and the *glpSTPQUV* genes of glycerol ABC transporters. Homologous gene clusters have been annotated in the genomes of 6 of the 15 type strains of the described *Pseudodesulfovibrio* species (Figure S8). However, the possibility of growth of these strains on glycerol in the presence of sulfates has not been studied, so the functionality of these gene clusters has not yet been confirmed.

It can be noted that homologous clusters of glycerol metabolism genes are also annotated in the genomes of some strains of the *Desulfovibrionales* order. In particular, such a gene cluster (Dbac_1434–Dbac_1446) was described for the sulfate-reducing bacterium *Desulfomicrobium baculatum* strain DSM 4028^T^. This strain, however, was not able to grow on glycerol as a substrate, as well as phylogenetically similar new strains isolated from an enrichment culture with glycerol and sulfate [70].

It was assumed that this is due to the presence of the mobile element protein gene (Dbac_1434) in front of the *glpK* gene, which indicates that the glycerol gene cluster might not be genetically stable and the strains cannot simply switch to glycerol as the substrate. In the genome of strain 5S69^T^ in the homologous location (V8V93_17540), the gene presumably of an acyltransferase family protein is annotated, as well as in the genomes of most species of the genus *Pseudodesulfovibrio* carrying a cluster of glycerol metabolism genes, except for *P. profundus* 500-1^T^. In the genome the latter species, the gene of the protein of the mobile element is also annotated in the homologous location. Apparently, the ability to use glycerol by sulfate reducers needs further study.

The strain 5S69^T^ can grow on alcohols, reducing sulfate. High sulfide production was observed on ethanol, but the strain also used methanol and exhibited weak growth on propanol. Ethanol oxidation occurs with alcohol dehydrogenase (ADH) (EC: 1.1.1.1) first to acetaldehyde, and then with aldehyde dehydrogenase (NAD+) (ALDH) (EC: 1.2.1.3) to acetate. This is confirmed by the presence in the genome of strain 5S69^T^ of several genes encoding ADH (V8V93_01960, 05810, 07300, 07315, 13095), as well as of the gene encoding ALDH (V8V93_11475). It can be noted that in the genomes of type strains of other *Pseudodesulfovibrio* species, the ALDH gene is annotated only in the strain SB368^T^ of the not validly published species “*P. pelocollis*” the ability of members of other species to oxidize ethanol via the ADH pathway remains therefore questionable.

The genes of specific enzymes responsible for methanol degradation to formaldehyde, such as methanol dehydrogenases (EC: 1.1.2.7; 1.1.2.10; 1.1.1.244), in the genome of strain 5S69^T^ have not been annotated; however, there is a possibility of methanol assimilation also via the ADH pathway. Presumably, methanol is oxidized using ADH to formaldehyde, and then using ALDH to formate. Previously, this ability was shown for other sulfate reducers, in particular, for type strains of *Desulfofundulus kuznetsovii* 17^T^ and *Desulfofundulus salinus* 435^T^, for which it was additional to the specific cobalt-dependent methyl transferase (MT) pathway in combination with the reducing acetyl-CoA pathway [71,72,73], as well as for another *Desulfofundulus kuznetsovii* strain, TROSR, for which the ADH pathway of methanol oxidation turned out to be the only one [74]. Presumably, methanol is oxidized using ADH to formaldehyde, and then using ALDH to formate. As was shown by proteomics results, the enzymes of this pathway were alcohol dehydrogenase (ADH) (EC: 1.1.1.1) encoded by one of several ADH-determining genes (Desku_2952), represented in the genome of *D. kuznetsovii* 17^T^, and aldehyde ferredoxin oxidoreductase (1.2.7.5) encoded by *aor* gene (Desku_02951). In the genome of strain 5S69^T^, homologous genes of alcohol dehydrogenase (V8V93_07300) and aldehyde ferredoxin oxidoreductase (V8V93_13365) were annotated, with 64 and 53% similarities of translated amino acid sequences, respectively. They probably encoded the enzymes of the ADH pathway of methanol assimilation. However, proteomic research of methanol consumption by strain 5S69^T^ is required for more accurate understanding of this process. Similarly, it is proposed to use the ADH pathway to assimilate propanol through propionaldehyde to propanoate. However, unlike formate, which is catabolized to CO_2_ by strain 5S69^T^, the genes for the degradation of acetate and propionate are not annotated in the genome, so these compounds can be accumulated in the culture medium.

#### 3.4.4. Pyruvate Fermentation

To ferment pyruvate to formate and acetyl-CoA, strain 5S69^T^ can use the enzyme pyruvate formate-lyase (EC: 2.3.1.54) encoded by the *pflD* gene (V8V93_03760). According to physiological data, the end products of pyruvate fermentation by the strain are acetate in the culture medium and CO_2_/H_2_ in the gas phase. According to the BlastCOALA service, in the 5S69^T^ genome are annotated the genes of two different enzymes, which catabolize formate to CO_2_. The enzyme NAD-dependent formate dehydrogenase (EC: 1.17.1.9), encoded by the *fdhAB* genes (V8V93_10940–10945; 06930–06925; 12995–13000; 18135–18140) has been characterized for bacteria of various taxonomic groups, including sulfate reducer *Desulfovibrio desulfuricans* ATCC 27774 [75]. The gene FDHB (V8V93_17305) encoded the enzyme formate dehydrogenase coenzyme F420-dependent (EC: 1.17.98.3), but was characterized only from methanogenic archaea [76]. Both enzymes can participate in formate-dependent H_2_ production in combination with hydrogenases, however, obtaining more accurate data on these processes requires special experiments.

For further fermentation of acetyl-CoA to acetate, the direct phosphate acetyltransferase-acetate kinase pathway is presumably used with the participation of the phosphate acetyltransferase (EC: 2.3.1.8) and acetate kinase (EC: 2.7.2.1) enzymes encoded by the *pta* (V8V93_05930) and *askA* (V8V93_05925, 15100) genes, respectively. In this case, acetate is accumulated in the culture medium. The genes of pyruvate fermentation enzymes to alcohol in the 5S69^T^ genome have not been identified.

#### 3.4.5. Sulfur Metabolism

**Figure 6:**
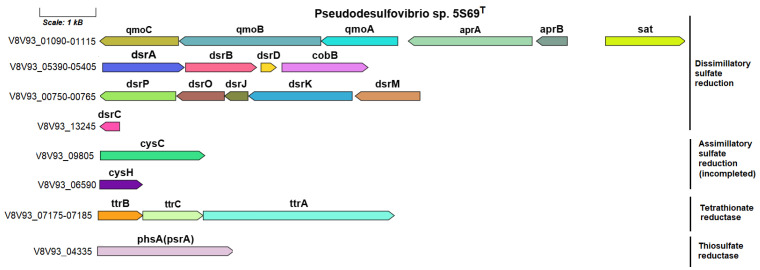
The genes presumably encoding sulfur metabolism in the genome of the strain 5S69^T^. Abbreviations: *sat*, sulfate adenylyltransferase; *aprAB*, adenylylsulfate reductase; *qmoABC*, proteins of adenylsulfate reductase-associated electron transfer complex; *dsrABD*, dissimilatory sulfite reductase; *dsrMKJOP*, [DsrC]-trisulfide reductase; *dsrC*, sulfur redox associated protein; *cobB*, protein similar to cobyrinic acid a,c-diamide synthetase clustered with dissimilatory sulfite reductase; *cysC*, adenylyl-sulfate kinase; *cysH*, phosphoadenosine phosphosulfate reductase; *ttrBCA*, tetrathionate reductase; *phsA*(*psrA*), thiosulfate reductase/polysulfide reductase. Scale bar, 1000 bp.

According to the sulfur metabolism KEGG map, the reduction of sulfate to sulfide by strain 5S69^T^ is carried out only via a dissimilatory process (Figure S9). An orthologous set of genes for dissimilatory sulfate reduction is present in the genomes of all strains of the genus *Pseudodesulfovibrio*. The genes determining sulfate reduction to silfite are represented by a cluster including the *sat* gene (V8V93_01115) encoding sulfate adenylyltransferase (EC: 2.7.7.4), *aprAB* genes (V8V93_01110–01105), coding two adenylylsulfate reductase subunits (EC: 1.8.99.2), as well as *qmoABC* genes (V8V93_01100–01090), coding the proteins of adenylsulfate reductase-associated electron transfer complex. The sulfur metabolism genes in the 5S69^T^ genome are shown in Figure 6.

The *dsrABD* gene cluster (V8V93_05390–05400), *dsrMKJOP* complex (V8V93_00770–00750), and the *dsrC* gene (V8V93_13245), coding further reduction of sulfite to sulfide, were similar to those of other sulfate-reducing bacteria. The *dsrC* gene (V8V93_13245), encoding sulfur redox associated protein, was localized in another part of the genome.

In addition, *ttrBCA* genes (V8V93_07175–07185) encoding the tetrathionate reductase enzyme, which reduces tetrathionate to thiosulfate, have been annotated in the 5S69^T^ genome. The ability to respire tetrathionate using molecular hydrogen as a reducing agent has been found in bacteria of various taxonomic groups, including sulfate reducers [77,78]. The genetic determinants of this process have been studied in detail for *Salmonella typhimurium* [79]. In addition to strain 5S69^T^, homologous Ttr clusters were annotated only in the genomes of strains *P. mercurii* ND132^T^ and ‘*P. thermohalotolerans*’ MCM B1480^T^, phylogenetically close to strain 5S69^T^. Further reduction of thiosulfate to sulfide, as well as the use of thiosulfate as an electron acceptor during growth, presumably occurs with the participation of the enzyme thiosulfate reductase/polysulfide reductase (EC: 1.8.5.5), encoded by the *phsA*(*psrA*) gene, annotated according to the BlastKOALA portal, in the genome of strain 5S69^T^ (V8V93_04345) and in genomes of other type strains of *Pseudodesulfovibrio* species.

Of the genes encoding enzymes of assimilatory sulfate reduction in the genome of strain 5S69^T^, as in the genomes of other *Pseudodesulfovibrio* spp., were present, the genes coding enzymes of the first stage of this process—the reduction of sulfate to sulfite, were, namely, the *cysC* gene (V8V93_09805) encoding adenylyl-sulfate kinase (EC: 2.7.1.25), and the *cysH* gene (V8V93_06590) encoding phosphoadenosine phosphosulfate reductase (EC: 1.8.1.8).

#### 3.4.6. Nitrogen Metabolism

The strain 5S69^T^ cannot use nitrate as an electron acceptor and as a nitrogen source for growth, which correlates with the absence of genes encoding assimilatory and dissimilatory nitrate reduction. According to the KEGG map of the nitrogen metabolism pathway, strain 5S69^T^ can carry out the process of molecular nitrogen fixation to form ammonium (Figure S10). The genome of the strain contains the *nifH* nitrogenase reductase gene as part of the gene cluster (V8V93_16905–16840), which also includes the genes of the nitrogenase subunits (*nifD* and *nifK*), a set of auxiliary genes *nifB_1*, *nifB_2*, *nifE*, *nifN*, as well as the genes that determine ferredoxin and proteins P-II–a family of regulators of nitrogen metabolism (Figure S11). Orthologous gene clusters have been annotated in a larger number of genomes of type strains of the species of the genus *Pseudodesulfovibrio* (except for *P. tunisiensis* DSM 19275^T^ and *P. nedwellii* SYK^T^), including the type species for the genus, *P. indicus* DSM 101483^T^. All these clusters are flanked by the *nifA* gene, which determines nitrogenase (molybdenum-iron)-specific transcriptional regulator, and the *nifV* gene, encoding homocitrate synthase. In the genome of the strain 5S69^T^ similar to the genomes of other *Pseudodesulfovibrio* spp., the *hcp* (V8V93_01945) gene was annotated, encoding hydroxylamine reductase (EC: 1.7.99.1), which reduce hydroxylamine to ammonium.

According to physiological data, strain 5S69^T^ is able to grow on glutamate, reducing sulfate. The genome of the strain contains the genes of enzymes of a possible glutamate conversion pathways: glutamate dehydrogenase (NAD) (EC: 1.4.1.2), encoded by the *gudB* gene (V8V93_14380), catalyzing the formation of ammonium and 2-oxoglutarate; glutamine synthetase (EC: 6.3.1.2), encoded by the *glnA* gene (V8V93_02895, 06195), catalyzing the incorporation of ammonium into glutamate with the formation of glutamine; as well as glutamate synthase (NADPH) large and small chains (EC: 1.4.1.13) encoded by the *glnB* and *glnDB* genes (V8V93_12290, 13680–13685), catalyzing a two-stage reaction of glutamate transformation to glutamine and 2-oxoglutarate. However, more accurate information about the functioning of these genes can be obtained by studying the activity of the corresponding enzymes.

#### 3.4.7. Oxidative and Osmotic Stress Response and Heavy Metal Resistance

While most sulfate-reducing bacteria are strict anaerobes, some strains are aerotolerant and may survive oxidative stress. Operation of a new oxidative stress defense system, characteristic only of anaerobes, has previously been shown for *Desulfovibrio vulgaris* strain Hildenborough. The major components of this system were the nonheme iron proteins, rubrerythrin (Rbr), and rubredoxin oxidoreductase (Rbo), the product of the *rbo* gene [80]. The gene structures containing these genes were different in different anaerobes. In the genome of strain 5S69^T^, a gene cluster was annotated (V8V93_00035–00060), containing the *rbo* (rubredoxin-oxygen oxidoreductase), *rub* (rubredoxin), *sorA* (superoxide reductase—EC 1.15.1.2), *rbo* (rubrerythrin), and *perE* (peroxide stress regulator PerR, FUR family). Presumably, this cluster encodes the oxidative stress defense system in strain 5S69^T^. Homologous gene clusters were present in the genomes of the type strains of all *Pseudodesulfovibrio* species. Moreover, the *trkAH* (V8V93_11220–11225) genes were annotated in the genome of 5S69^T^. They encode the Trk potassium uptake system protein, which enhances the biofilm formation and cell membrane stability under hyperosmotic conditions [81].

Decreasing the concentrations of heavy metals by the enzymatic systems for effusion removal of metal ions out of the cells is a usual approach of many bacteria to heavy metal resistance. In the genome of strain 5S69^T^, the *znuABC* genes (V8V93_05370, 08285–08290) were annotated, encoding a high-affinity transporter specialized for transporting zinc ions as part of a system for metal ion homeostasis in bacteria [82]. It is regulated by the V8V93_08285 (Zur) protein, encoded by the *zur* gene (V8V93_05375). The mechanism of resistance to chromate, probably determined by the product of the *chrA* gene (V8V93_01340), appears to be based on the active efflux of chromate driven by the membrane potential [83].

The genome of strain 5S69^T^ was searched for the genes coding formation of the main osmoprotectors—betaine and ectoine, which are included in the Glycine, serine, and threonine metabolism pathway in the KEGG database. As a result, a cluster of *betAB* genes (V8V93_12115–12120) was identified that determines the enzymes of choline oxidation to betaine aldehyde (choline dehydrogenase EC: 1.1.99.1) and further to betaine (betaine-aldehyde dehydrogenase, EC: 1.2.1.8), as well as glycine betaine transport system genes, which indicates the potential for the synthesis of betaine by the strain and its use as an osmoprotector in highly saline groundwater. Homologous clusters have been found in the genomes of other *Pseudodesulfovibrio* spp. At the same time, ectoine synthesis genes in the genomes of type strains of this genus have not been annotated.

#### 3.4.8. Hydrogenase Genes

The strain 5S69^T^ supports complex hydrogen metabolism due to the presence in its genome of three gene clusters encoding hydrogenases, differing both in structure and intracellular localization. The *echABCDEF* cluster (V8V93_06595–06620) presumably encodes the multisubunit energy-conserving, membrane-bound Ech-[NiFe]-hydrogenase (ferrodoxin), which catalyzes the reversible reaction of proton reduction to H_2_ formation with a ferredoxin site and is inherent in some bacteria and archaea [84]. The model object for studying this enzyme is a sulfate reducer *Desulfovibrio fructosovorans* JJ^T^ [85], which was later transferred to the genus *Solidesulfovibrio* (*S. fructosivorans*) [28]. According to the BlastCOALA portal, gene clusters homologous to the *echABCDEF* operon of the JJ^T^ strain, having 46–95% similarity of translated amino acid sequences, were identified in the genome of strain 5S69^T^ and other type strains of the genus *Pseudodesulfovibrio*, which suggests an important role of Ech hydrogenases in H_2_ metabolism for members of this genus.

It should be noted that in the genome of the sulfate reducer *Nitratidesulfovibrio vulgaris* strain Hildenborough, in addition to the Ech hydrogenase genes, the *CooMKLXUH* gene structure encoding another membrane-bound multisubunit Coo-[NiFe]-hydrogenase (carbon monoxide-induced hydrogenase) [86] was identified, which in the genomes of strains 5S69^T^ and JJ^T^ was not revealed, although it was assumed that this enzyme is essential for the growth in a lactate-sulfate medium [87], which strain 5S69^T^ is also capable of. Of the members of the genus *Pseudodesulfovibrio*, the operon homologous to *CooMKLXUH* is annotated only in the genome of the *P. piezophilus* C1TLV30^T^.

Strain 5S69^T^ also contains soluble hydrogenases of the [FiFe]- and [NiFe]-types localized in the periplasm, but no gene was found for a [NiFeSe]-enzyme. One of them is presumably HynAB-type cytochrome-c3 [NiFe] hydrogenase (EC: 1.12.2.1), which is encoded by the genes of the large and small subunits of *hynAB* (V8V93_08215–08220). The enzyme catalyzes a reversible oxidation-reduction reaction of hydrogen using cytochrome-c3, depending on the growth conditions: it oxidizes hydrogen in the presence of sulfate and produces hydrogen during the fermentation of pyruvate or lactate [88]. Another soluble enzyme, periplasmic heterodimeric Hyd-[FeFe] hydrogenase (EC: 1.12.7.2), in the 5S69^T^ genome is also encoded by the genes of the large and small subunits *hydAB* (V8V93_01515–01520). This hydrogenase also performs a reversible oxidation-reduction reaction of hydrogen, but with the participation of ferredoxin. It is assumed that Hyd-[FeFe] periplasmic hydrogenase is involved in H_2_ oxidation at high H_2_ partial pressure in keeping with its low affinity for H_2_ and its high specific activity [89]. The *hynAB* and *hydAB* genes are annotated in the complete genomes of all type strains of the genus *Pseudodesulfovibrio*. In addition, the 5S69^T^ genome contains auxiliary *hypAB* genes encoding [NiFe]-hydrogenase nickel incorporation protein, *hypCDE* genes encoding [NiFe]-hydrogenase metallocenter assembly protein, and *hypEF* genes encoding [FeFe]-hydrogenase maturation protein.

#### 3.4.9. Mercury Methylation Genes

A number of SRB of the genus *Pseudodesulfovibrio* are able to methylate mercury compounds to form the toxic organic pollutant methylmercury (CH_3_Hg^+^) [26,29]. The ability to methylate mercury has been studied in the most detail for the type strain *Pseudodesulfovibrio* (formerly *Desulfovibrio*) *mercurii* NOD132^T^ [29,90], phylogenetically close to strain 5S69^T^. According to analysis of the genomes of mercury-methylating and non-methylating bacteria, the genetic determinants of this process were determined [91]. In the genomes of Hg methylators the *hgcA* gene is present, encoding a putative mercury methylation corrinoid protein, and an additional, downstream located *hgcB* gene, encoding a ferredoxin-like protein. In this work, we did not experimentally test the ability to methylate mercury by strain 5S69^T^. However, a comparison of the nucleotide sequences of the *hgcAB* genes (V8V93_15395–15400) annotated in the 5S69^T^ genome showed their 77% homology with the genes of *P. mercurii* NOD132^T^, which indicates the potential ability of strain 5S69^T^ to methylate mercury. The resistance of the 5S69^T^ strain to mercury ions is presumably provided by the enzyme mercuric ion reductase (EC: 1.16.1.1) encoded by the *merA* gene (V8V93_12795).

## 4. Conclusions

The data obtained indicate that the oil reservoirs of Tatarstan (Russia) are inhabited by sulfate-reducing bacteria participating in the biogeochemical cycles of carbon and sulfur in this subsurface environment. Phenotypic and genomic characteristics of the sulfate-reducing strain 5S69^T^ isolated from injection water collected at the Vostochno-Anzirskoe oil field were studied and its taxonomic affiliation to the genus *Pseudodesulfovibrio* was determined. The strain 5S69^T^ is distinguished from its closest relatives of the genus *Pseudodesulfovibrio* by a set of physiological features, including its ability to use malate, methanol, ethanol, glycerol, and fructose as carbon and electron donors and its inability to grow on glucose, sucrose, and lactose and by nitrate reduction (Table 1). The strain has a rare ability to grow on methanol, which, among the members of this genus, was found only in *Pseudodesulfovibrio tunisiensis* and “P*seudodesulfovibrio cashew*”. The results of phenotypic studies, phylogenetic analysis of the 16S rRNA gene sequence and 120 conserved single copy proteins, as well as values of genomic indexes (ANI, AAI, and dDDH) between strain 5S69^T^ and the type strains of *Pseudodesulfovibrio* species demonstrated that strain 5S69^T^ constituted a novel species within the genus *Pseudodesulfovibrio*, for which the name *Pseudodesulfovibrio methanolicus* sp. nov. is proposed. Although the strain was isolated from injection water, i.e., a mixture of fresh river and formation water, its physiological characteristics—the ability to grow at high salinity, at reservoir temperature, and on media with alcohols or H_2_/CO_2_ in the presence of acetate—indicate adaptation to the conditions of the oil reservoir and its subsurface origin. The description of the species *Pseudodesulfovibrio methanolicus* sp. nov. is given in the protologue (Table 2).

## Figures and Tables

**Table 1:** Differential characteristics of sulfate-reducing strain 5S69^T^ and type strains of phylogenetically most closely related *Pseudodesulfovibrio* species.

Characteristic	1	2	3	4	5
Type strain	5S69^T^	J2^T^	BerOc1^T^	ND132^T^	MCM B-1480^T^
Motility	+	+	+	+	+
NaCl, min-opt-max, % (*w*/*v*)	0.2–(2–4)–6	0.2–2.5–6.0	0.2–1.5–4.0	0–2–3	1–3–6
Temperature, min-opt-max, °C	15–(23–28)–37	9–(30–35)–40	25–30–35	20–32–37	20–37–60
pH, min-opt-max	4.6–6.5–8.6	5.0–(6.5–7.0)–8.0	–(6.0–7.4)–	6.8–7.8–8.2	6–7–8
Electron donor with sulfate:					
H_2_/CO_2_	+ *	+	+	+ *	ND
Formate	+	+	−	+ *	+
Succinate	+	ND	−	−	+
Fumarate	+	−	+	+	+
Citrate	W	ND	−	ND	−
Malate	+	+	−	−	ND
Benzoate	−	ND	−	ND	ND
Methanol	+	ND	−	−	ND
Ethanol	+	−	W	−	ND
Glycerol	+	ND	−		ND
Glucose	−	ND	−	−	+
Sucrose	−	ND	−	ND	+
Fructose	+	ND	−	ND	+
Lactose	−	ND	−	ND	+
Galactose	W	ND	−	ND	+
Electron acceptor:					
Sulfite, thiosulfate	+	+	+	+	+
Elemental sulfur	+	−	−	ND	−
Nitrate	−	W	−	−	+
Fermentation of:					
Lactate	−	+	−	−	+
Fumarate	+	−	−	+	+
Genome size (Mb)	4.16	3.96	4.1	3.86	3.87
Genomic G + C content (mol %)	63.0	63.5	64.0	65.2	60.39
Major cellular fatty acids	*i*-C_15:0_, *ai*-C_15:0_, C_16:0_	*i*-C_15:0_, *ai*-C_15:0_, *i*-C_17:1_ ω9c, *i*-C_17:0_	C_18:0_, *ai*-C_15:0_, C_16:0_, C_18:1_ ω7	*i*-C_15:0_, *ai*-C_15:0_, *i*-C_17:0_, *i*-C_17:1_ ω9c	*i*-C_15:0_, *ai*-C_15:0_, C_16:0_, *ai*-C_17:0_
Isolation source	Hydrocarbon reservoir	Deep hydrothermal vent	Brackish sediments	Estuarine sediment	Hydrocarbon reservoir
Relatedness to strain 5S69^T^					
Identity of 16S rRNA (%)	100	98.0	99.5	98.9	98.4
ANI (%)	100	83.7	90.0	87.7	84.6
AAI (%)	100	80.9	90.0	87.2	84.8
dDDH (%)	100	26.2	40.3	34.5	28.5

Taxa: 1, *P. methanolicus* 5S69^T^ (this study); 2, *P. indicus* J2^T^ [24]; 3, *P. hydrargyri* BerOc1^T^ [26]; 4, *P. mercurii* ND132^T^ [29]; 5, ‘*P. thermohalotolerans’* MCM B-1480^T^ [32]. All taxa were able to ferment pyruvate and utilize lactate and pyruvate, but not acetate as substrates for sulfate reduction. Designations: ‘+’, positive reaction; (W), weakly positive reaction; ‘−’, negative reaction; ND, not determined. * Growth was observed in the presence of acetate. Data were retrieved from respective reference; the genomic characteristics of some strains were obtained from GenBank.

**Table 2:** Protologue description of *Pseudodesulfovibrio methanolicus* sp. nov.

Parameter	*Pseudodesulfovibrio methanolicus* sp. nov.
Genus name	*Pseudodesulfovibrio*
Species name	*Pseudodesulfovibrio methanolicus*
Species status	sp. nov.
Species etymology	me.tha.no’li.cus. N.L. neut. n. *methanol*, methanol; N.L. masc. adj. *methanolicus*, pertaining to methanol
Designation of the Type Strain	5S69^T^
Strain Collection Numbers	VKM B-3653^T^ = KCTC 25499^T^ = UQM 41509^T^
Genome accession number	GCF_037094465.1
Genome status	Complete
Genome size	4.16 Mb
GC mol%	63.0
16S rRNA gene accession nr.	PP792559.1
Description of the new taxon and diagnostic traits	The cells are straight or slightly curved rods, motile due a single flagellum, stained Gram-negative, and have cell wall structure typical of Gram-negative bacteria. Growth is observed in the presence of 0.2–6.0% (*w*/*v*) NaCl (optimum, 2.0–4.0% NaCl), at pH 4.6–8.6 (optimum, pH 6.5), and at 15–37 °C (optimum, 23–28 °C) under sulfate-reducing conditions. Strictly anaerobic. Reduces sulfate to sulfide in media with formate, lactate, pyruvate, malate, fumarate, succinate, methanol, ethanol, glycerol, fructose, and yeast extract as carbon and energy sources; weak growth observed on glutamate, citrate, propanol, galactose, and mannose, but does not use acetate, propionate, butyrate, glycine, L-serine, ornithine, glucose, lactose, sucrose, and benzoate. Hydrogen is utilized as electron donor for sulfate reduction in the presence of acetate as a carbon source. Lactate is oxidized with the production of acetate. Fermentative growth is observed with pyruvate, but lactate is not fermented. It uses sulfate, thiosulfate, sulfite, and fumarate as electron acceptors in the presence of lactate, but does not use nitrate. The predominant cellular fatty acids are *iso*-C_15:0_, *anteiso*-C_15:0_, and C_16:0_. The major polar lipids are phosphatidylethanolamine, diphosphatidylglycerol, phosphatidylglycerol, glycolipid, and phosphatidylserine. The major respiratory quinone is menaquinone MK-6(H_4_). The genome size of the type strain is 4.16 Mb with a genomic G + C content of 63.0 mol%. The type strain, 5S69^T^ (VKM B-3653^T^ = KCTC 25499^T^ = UQM 41509^T^), was isolated from the Vostochno-Anzirskoe oil field, in Yelabuzhsky district, Tatarstan, Russian Federation. The GenBank/EMBL/DDBJ accession number for the 16S rRNA gene sequence is PP792559.1 and the genomic assembly accession number is GCF_037094465.1.
Country and region of origin	Russian Federation, Tatarstan, Yelabuzhsky district
Date of isolation	2018
Source of isolation	A mixture of injection fresh river water and production water from the Vostochno-Anzirskoe oil field
Sampling date	June 2016
Latitude, Longitude	55°66′69″ N, 51°49′84.00″ E
Depth (meters below sea level)	1585
Number of strains in study	1
Information related to the Nagoya Protocol	Not applicable

## Data Availability

The whole-genome shotgun project of strain 5S69^T^ has been deposited at DDBJ/EMBL/GenBank under the accession GCF_037094465.1, and it is the first version described in this paper.

## Supplementary Materials

The following supporting information can be downloaded at: https://www.mdpi.com/article/10.3390/biology13100800/s1, Figure S1: Growth profiles of strain 5S69^T^ incubated in the lactate-sulfate medium at various temperatures (a), NaCl concentrations (g·L^−1^) (b), and pH (c) for 14 days; Figure S2: Polar lipids profiles from the strains 5S69^T^ and *Desulfovibrio desulfuricans* VKM B-1799^T^ (b). The components on the two-dimensional thin layer chromatograms were visualized by staining with 5% sulfuric acid in ethanol and heating at 180 °C for 15 min. Abbreviations: PE, phosphatidylethanolamines; DPG, diphosphatidylglycerols; PG, phosphatidylglycerols; GL, glycolipids; PL, phospholipids; PS, phosphatidylserines; LPE, lysophosphatidylethanolamines; GPL, glycophospholipids; Figure S3: Identification of polar lipids from the strains 5S69^T^ (A) and *Desulfovibrio desulfuricans* B-1799^T^ (B). The components were visualized by molybdenum blue (a); α-naphthol (b); ninhydrin (c); and Dragendorff’ reagent (d). Abbreviations as in Figure S2; Figure S4: KEGG-map of fructose and mannose metabolism pathways based on the genome analysis of the strain 5S69^T^. The presumptive pathways of fructose and mannose metabolism are highlighted in red. The enzymes annotated in the genome are highlighted in green in Figures S4–S7, S9 and S10; Figure S5: KEGG-map of galactose metabolism pathways based on the genome analysis of the strain 5S69^T^. The presumptive pathways of galactose metabolism are highlighted in red; Figure S6: KEGG-map of pyruvate metabolism pathways based on the genome analysis of the strain 5S69^T^. The pathways of pyruvate oxidation are highlighted in pink. The pathways of lactate, malate, fumarate, and succinate oxidation are highlighted in red; Figure S7: KEGG-map of other carbon fixation pathways based on the genome analysis of the strain 5S69^T^. The reversed phosphate acetyltransferase-acetate kinase pathway and conversion acetyl-CoA to pyruvate is highlighted in red; Figure S8: The genes presumably encoding the enzymes of glycerol metabolism pathway in the genome of the strain 5S69^T^ and other sulfate-reducing bacteria. Abbreviations: 1, acyltransferase family protein; 2, mobile element protein (trasposase); *glpK*, glycerol kinase; *hbrBCA*, heterodisulfide reductase-like protein; *glpBA*, anaerobic glycerol-3-phosphate dehydrogenase; *glpR*, glycerol-3-phosphate regulon repressor; *glpSTPQUV*, glycerol ABC transporters. Scale bar, 1000 bp; Figure S9: KEGG-map of sulfur metabolism pathways based on the genome analysis of the strain 5S69^T^. The dissimilatory sulfate reduction pathway is highlighted in pink. Tetrathionate and thiosulfate reduction pathways are highlighted in red; Figure S10: KEGG-map of nitrogen metabolism pathways based on the genome analysis of the strain 5S69^T^. The nitrogen fixation pathway is highlighted in pink. Glutamate and hydroxylamine metabolism pathways are highlighted in red; Figure S11: Organization of gene clusters presumably encoding nitrogen fixation enzymes in the genome of strain 5S69^T^ and other type strains of *Pseudodesulfovibrio* species. Abbreviations: *nifH*, nitrogenase (molybdenum-iron) reductase and maturation protein; *nifDK*, nitrogenase (molybdenum-iron) alpha and beta subunits*; nifB_1-2*, nitrogenase FeMo-cofactor synthesis FeS core scaffold and assembly protein; Fd, ferredoxin; P-II, nitrogen regulatory proteins; *nifN*, nitrogenase FeMo-cofactor scaffold and assembly protein; *nifE*, nitrogenase iron-molybdenum cofactor biosynthesis protein; *nifA*, nitrogenase (molybdenum-iron)-specific transcriptional regulator; *nifV*, homocitrate synthase; Table S1: Cellular fatty acid composition of strain 5S69^T^ and type strain of *Desulfovibrio desulfuricans* B-1799^T^; Table S2: The list of unique genes in the genome of strain 5S69^T^.
