# Supplementary material for: Phenotypic and Genomic Characterization of a Sulfate-Reducing Bacterium Pseudodesulfovibrio methanolicus sp. nov. Isolated from a Petroleum Reservoir in Russia
Source: Biology (Basel). 2024 Oct 7;13(10):800. doi: 10.3390/biology13100800 (PMC11505543; doi:10.3390/biology13100800)
Supplement: Supplementary file 1 [file biology-13-00800-s001.zip › Supplementary Materials.pdf]

biology

MDPI

Supplementary Materials

Phenotypic and Genomic Characterization of a Sulfate-Reducing Bacterium *Pseudodesulfovibrio methanolicus* sp. nov. Isolated from a Petroleum Reservoir in Russia

Salimat K. Bidzhieva [1], Tatyana P. Tourova [1], Vitaly V. Kadnikov [2], Salima R. Samigullina [1], Diyana S. Sokolova [1], Andrey B. Poltaraus [3], Alexander N. Avtukh [4], Vera M. Tereshina [1], Alexey V. Beletsky [2], Andrey V. Mardanov [2], and Tamara N. Nazina [1,*]

[1] Winogradsky Institute of Microbiology, Research Center of Biotechnology, Russian Academy of Sciences, Moscow 119071, Russia; salima.bidjieva@gmail.com (S.K.B.); tptour@rambler.ru (T.P.T.); samigullinasalimar@gmail.com (S.R.S.); sokolovadiyana@gmail.com (D.S.S.); v.m.tereshina@inbox.ru (V.M.T.); nazina@inmi.ru (T.N.N.)

[2] Institute of Bioengineering, Research Center of Biotechnology, Russian Academy of Sciences, Moscow 119071, Russia; vkadnikov@bk.ru (V.V.K.); mortu@yandex.ru (A.V.B.); mardanov@biengi.ac.ru (A.V.M.)

[3] Engelhardt Institute of Molecular Biology, Russian Academy of Sciences, 119991 Moscow, Russia; abpolt@gmail.com (A.B.P.)

[4] Skryabin Institute of Biochemistry and Physiology of Microorganisms, Russian Academy of Sciences, Pushchino Scientific Center for Biological Research of the Russian Academy of Sciences, Pushchino, Moscow region 142290, Russia; avtukh@rambler.ru

* Correspondence: nazina@inmi.ru; Tel.: +7-499-135-0341

This file includes:

Figures S1 to S11

Table S1

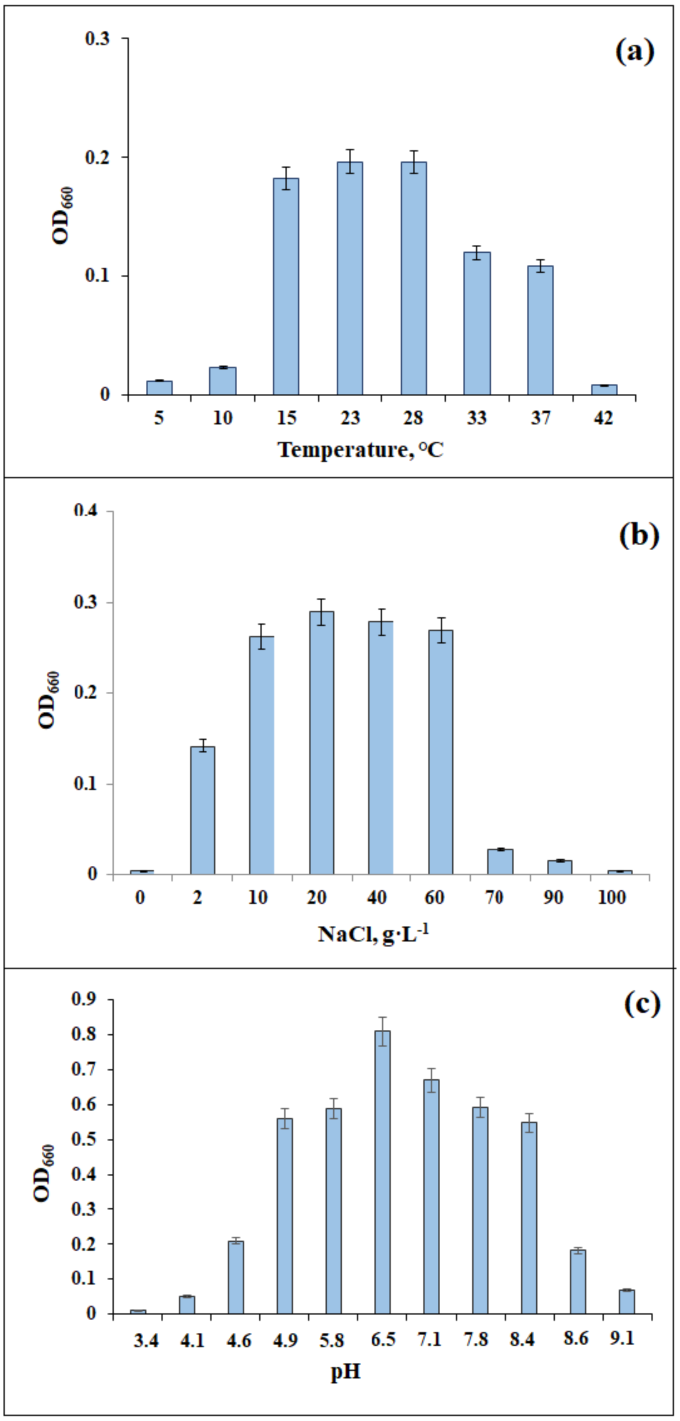

Figure S1. Growth profiles of strain 5S69^T incubated in the lactate-sulfate medium at various temperatures (a), NaCl concentrations ($g \cdot L^{-1}$) (b), and pH (c) for 14 days.

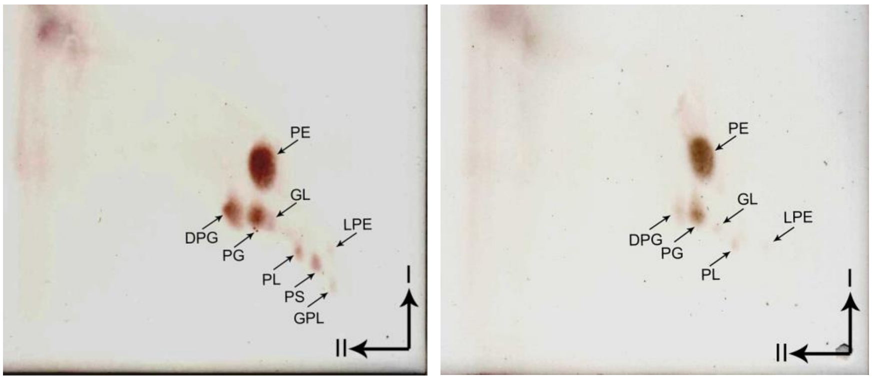

(a) **(b)**

Figure S2. Polar lipids profiles from the strains 5S69^{T} and *Desulfovibrio desulfuricans* VKM B-1799^{T} (b). The components on the two-dimensional thin layer chromatograms were visualized by staining with 5% sulfuric acid in ethanol and heating at 180 °C for 15 min. Abbreviations: PE, phosphatidylethanolamines; DPG, diphosphatidylglycerols; PG, phosphatidylglycerols; GL, glycolipids; PL, phospholipids; PS, phosphatidylserines; LPE, lysophosphatidylethanolamines; GPL, glycophospholipids.

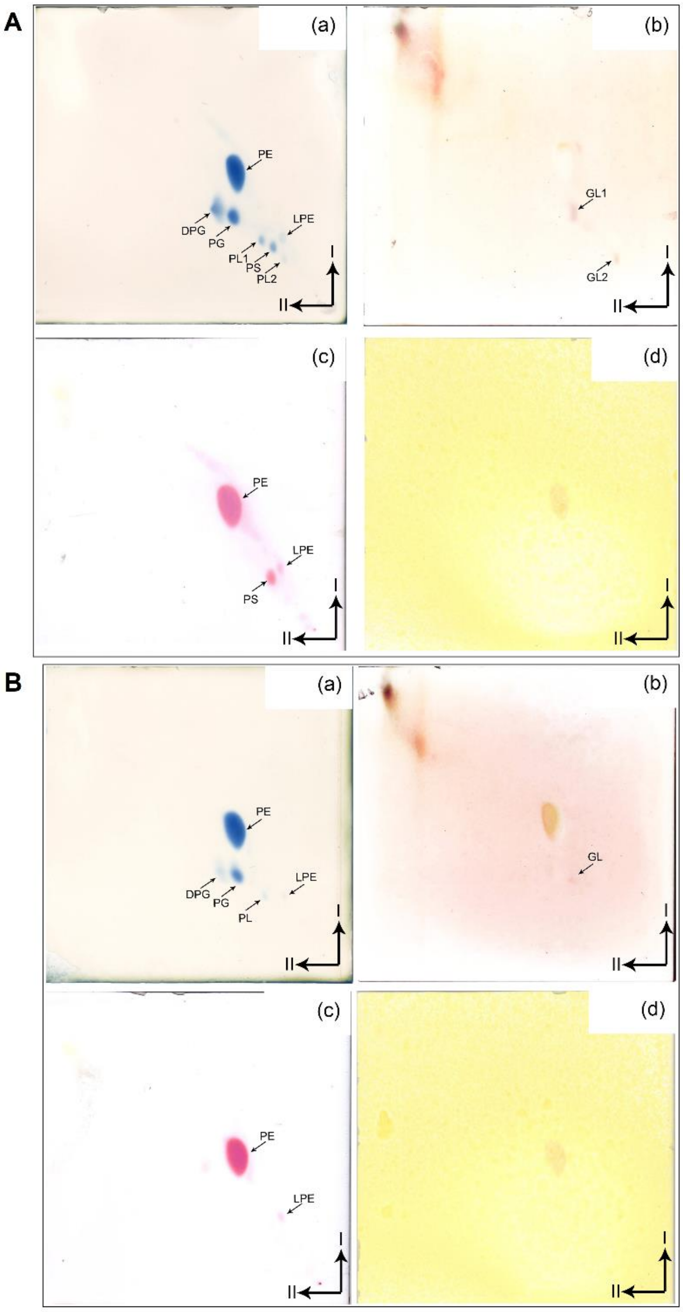

Figure S3. Identification of polar lipids from strains 5S69^{T} (A) and *Desulfovibrio desulfuricans* B-1799^{T} (B). The components were visualized by molybdenum blue (a); α-naphthol (b); ninhydrin (c); and Dragendorff′ reagent (d). Abbreviations as in Figure S2.

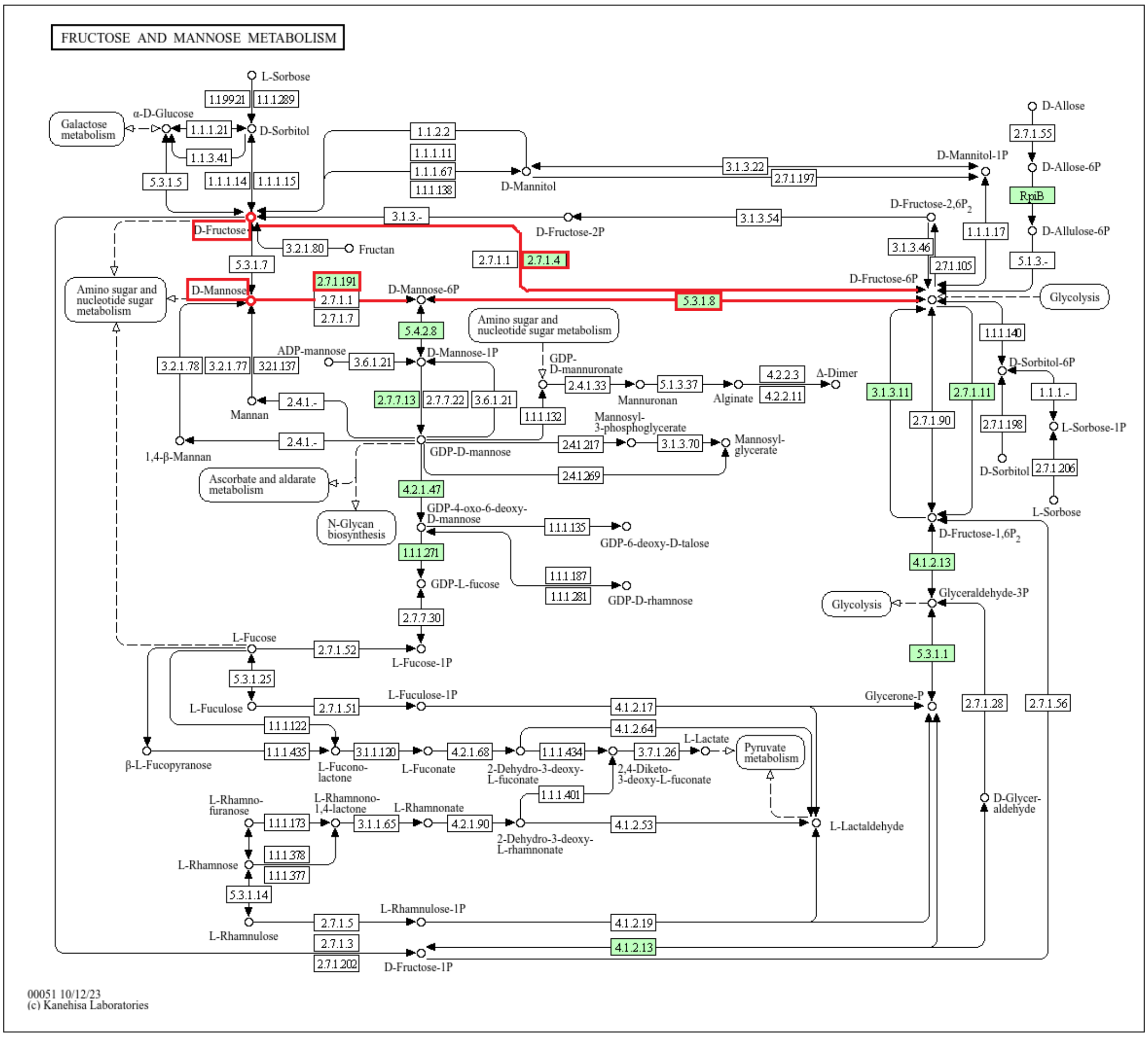

Figure S4. KEGG-map of fructose and mannose metabolism pathways based on the genome analysis of the strain 5S69ᵀ. The presumptive pathways of fructose and mannose metabolism are highlighted in red. The enzymes annotated in the genome are highlighted in green in Figures S4-S7, S9, and S10.

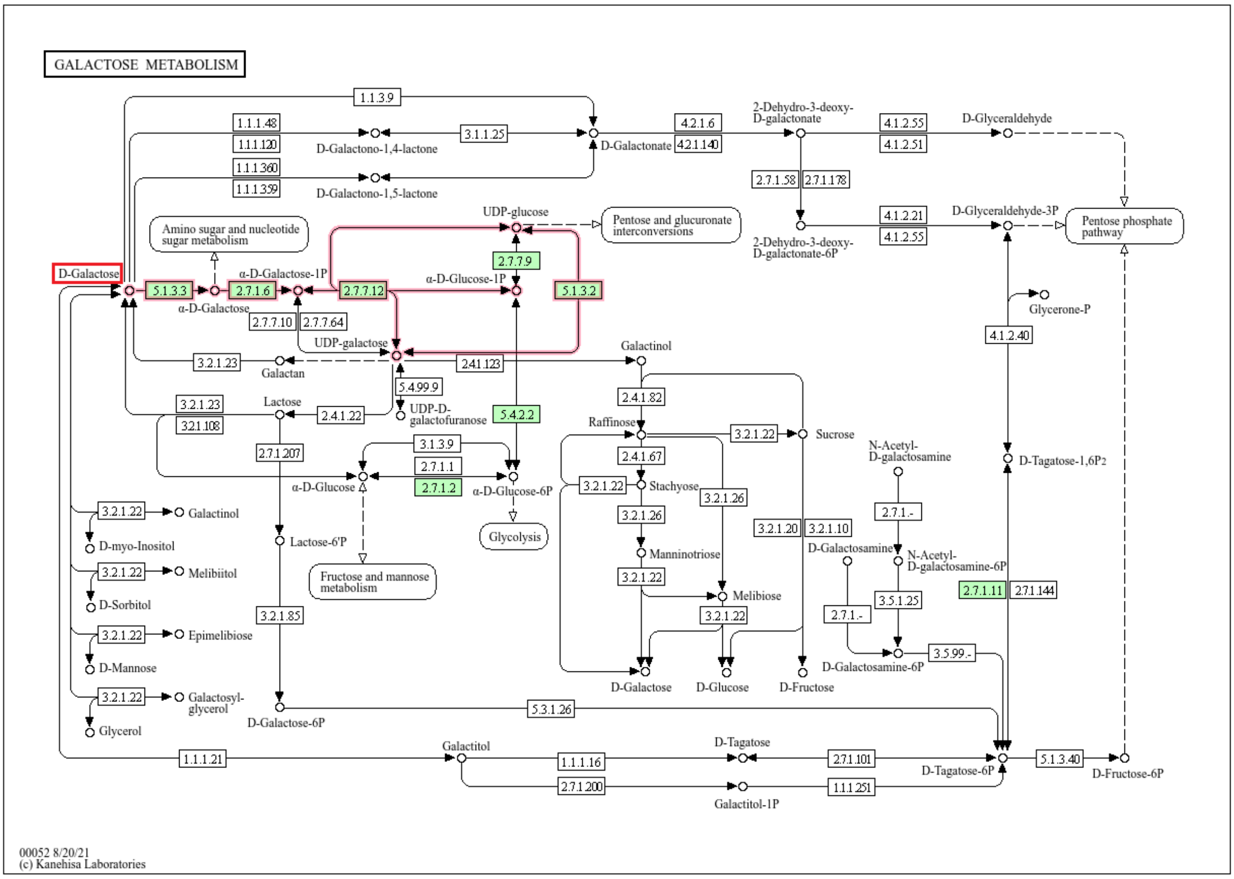

Figure S5. KEGG-map of galactose metabolism pathways based on the genome analysis of the strain 5S69[T]. The presumptive pathways of galactose metabolism are highlighted in red.

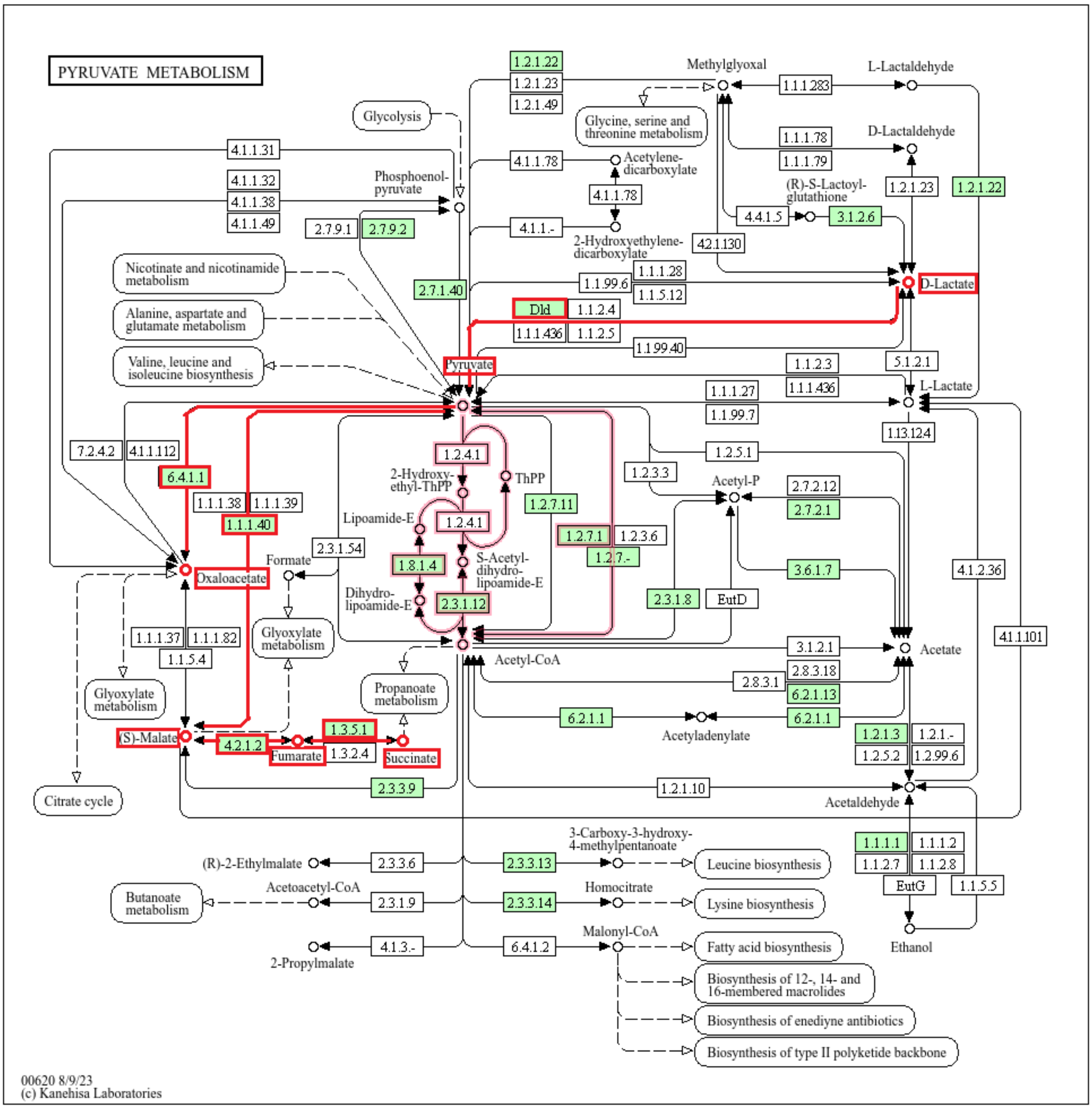

Figure S6. KEGG-map of pyruvate metabolism pathways based on the genome analysis of the strain 5S69^{T}. The pathways of pyruvate oxidation are highlighted in pink. The pathways of lactate, malate, fumarate, and succinate oxidation are highlighted in red.

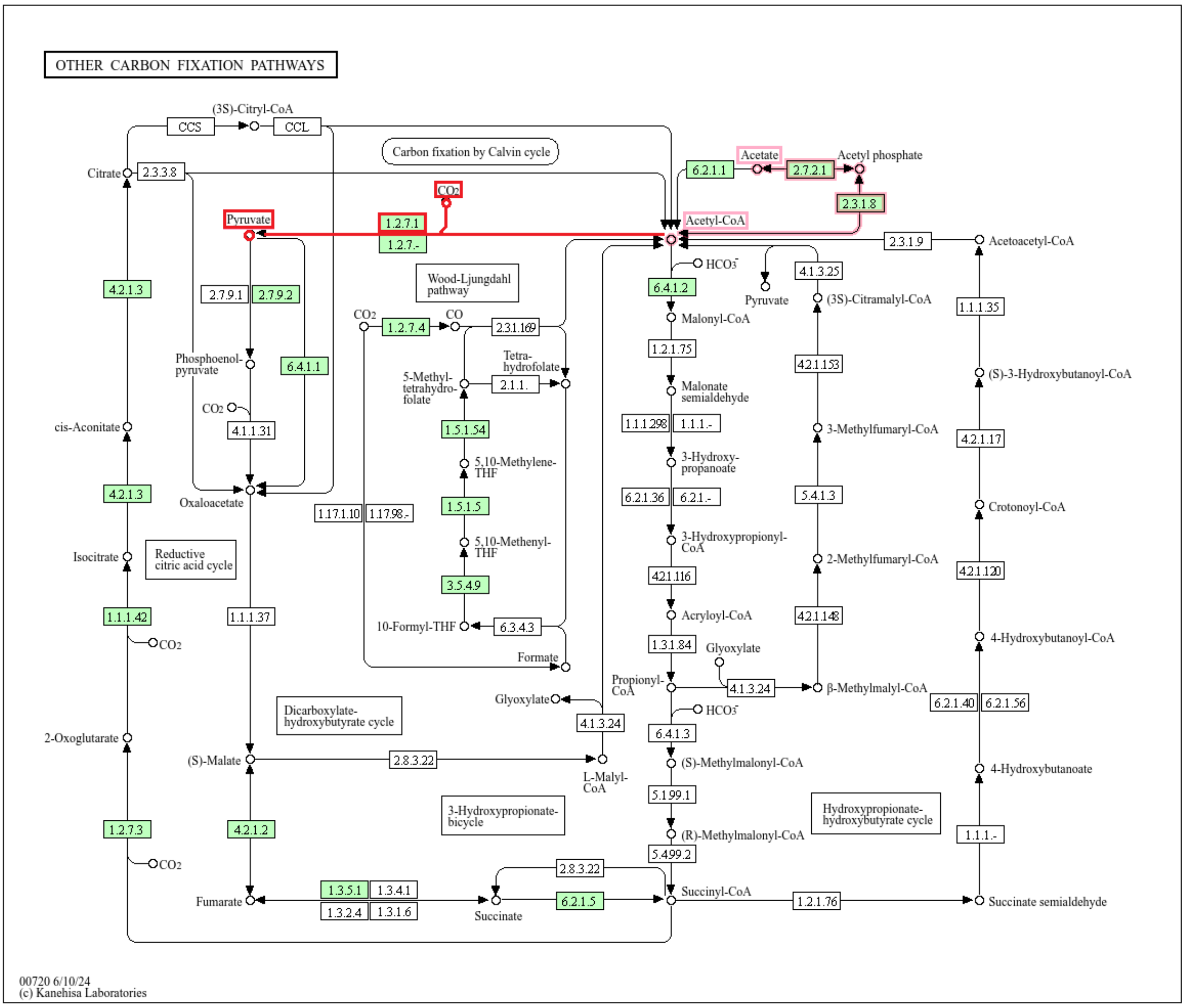

Figure S7. KEGG-map of other carbon fixation pathways based on the genome analysis of the strain 5S69[T]. The reversed phosphate acetyltransferase-acetate kinase pathway and conversion acetyl-CoA to pyruvate is highlighted in red.

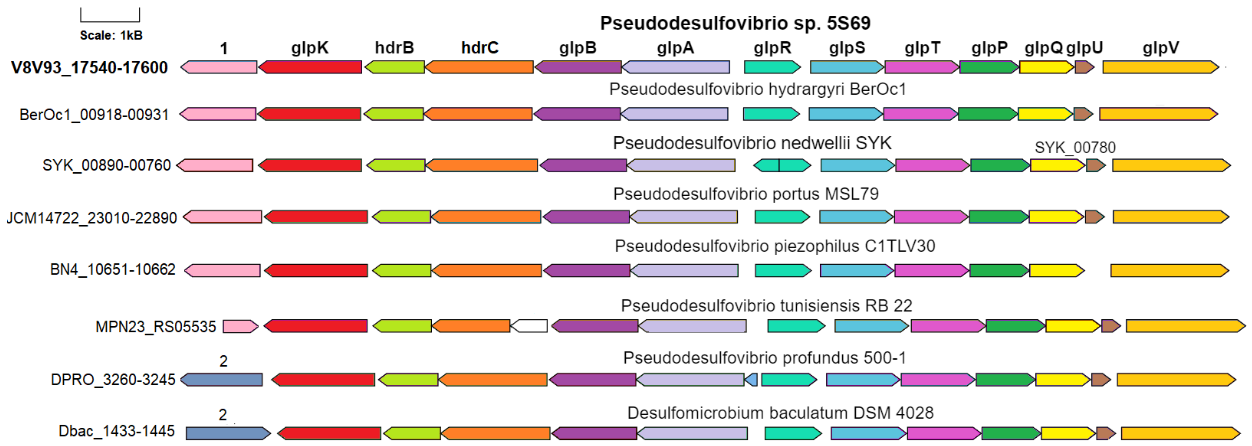

Figure S8. The genes presumably encoding the enzymes of glycerol metabolism pathway in the genome of the strain 5S69^{T} and other sulfate-reducing bacteria. Abbreviations: 1, acyltransferase family protein; 2, mobile element protein (trasposase); *glpK*, glycerol kinase; *hbrBCA*, heterodisulfide reductase-like protein; *glpBA*, anaerobic glycerol-3-phosphate dehydrogenase; *glpR*, glycerol-3-phosphate regulon repressor; *glpSTPQUV*, glycerol ABC transporters. Scale bar, 1000 bp.

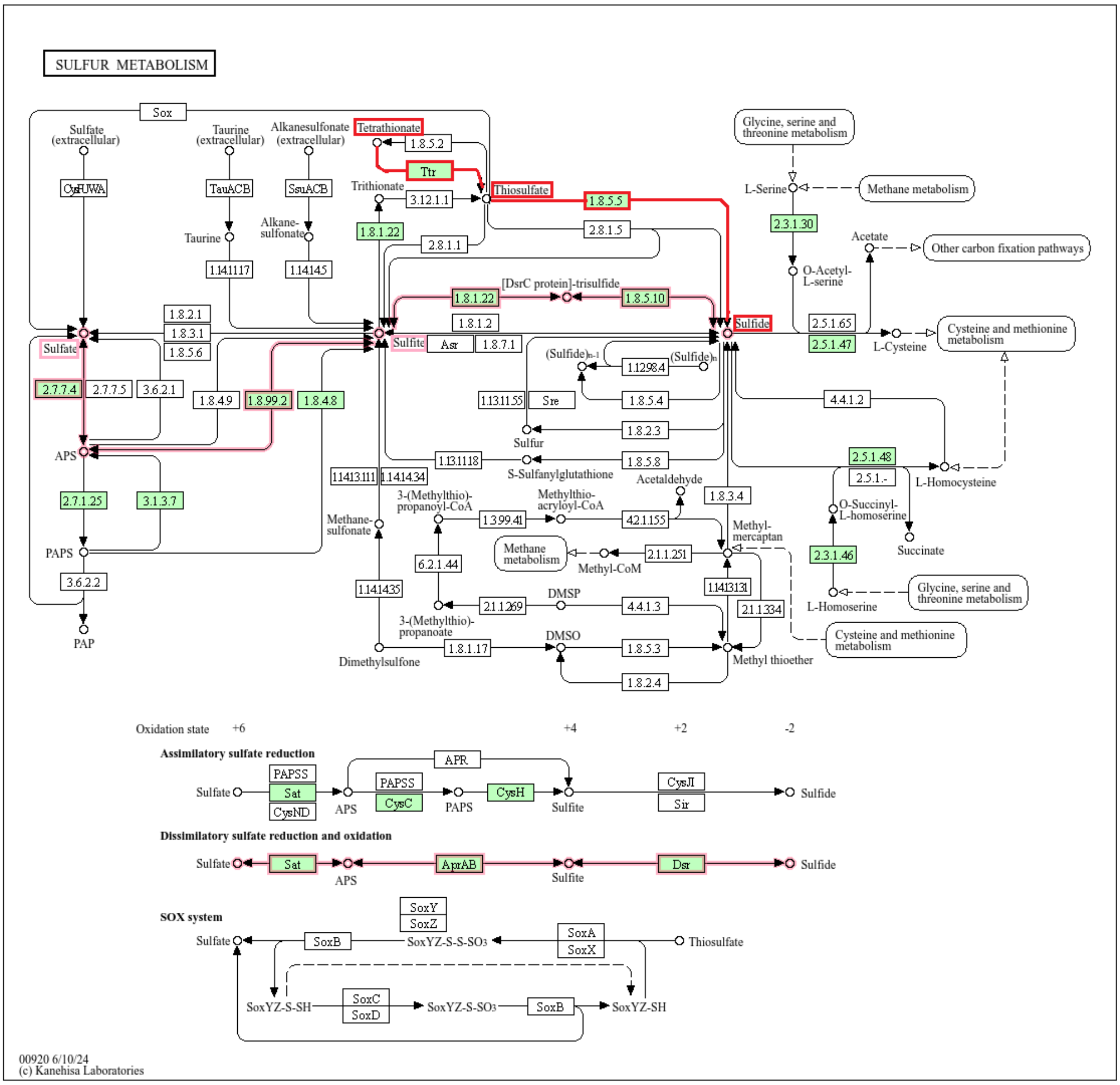

Figure S9. KEGG-map of sulfur metabolism pathways based on the genome analysis of the strain 5S69[T]. The dissimilatory sulfate reduction pathway is highlighted in pink. Tetrathionate and thiosulfate reduction pathways are highlighted in red.

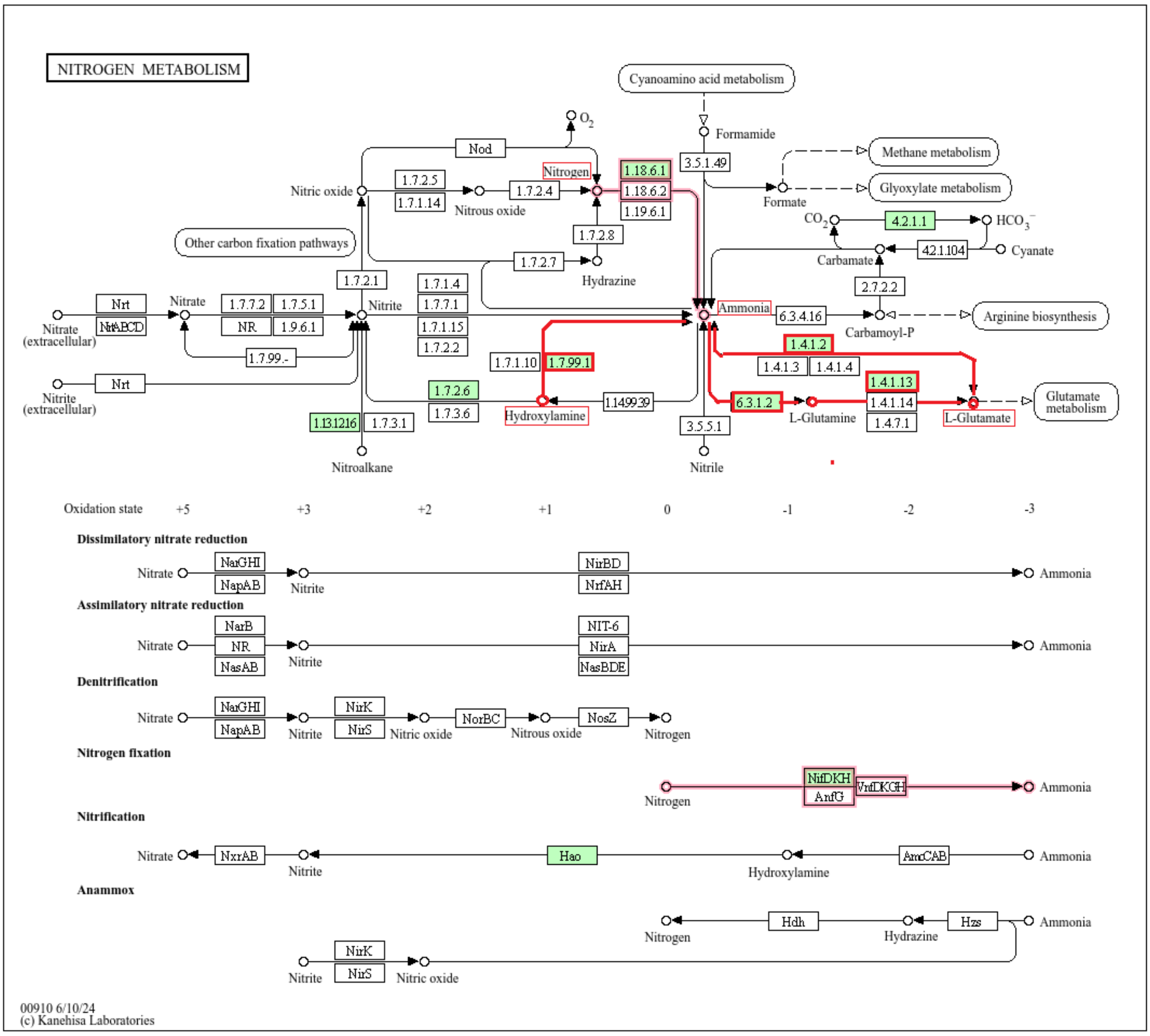

Figure S10. KEGG-map of nitrogen metabolism pathways based on the genome analysis of the strain 5S69^{T}. The nitrogen fixation pathway is highlighted in pink. Glutamate and hydroxylamine metabolism pathways are highlighted in red.

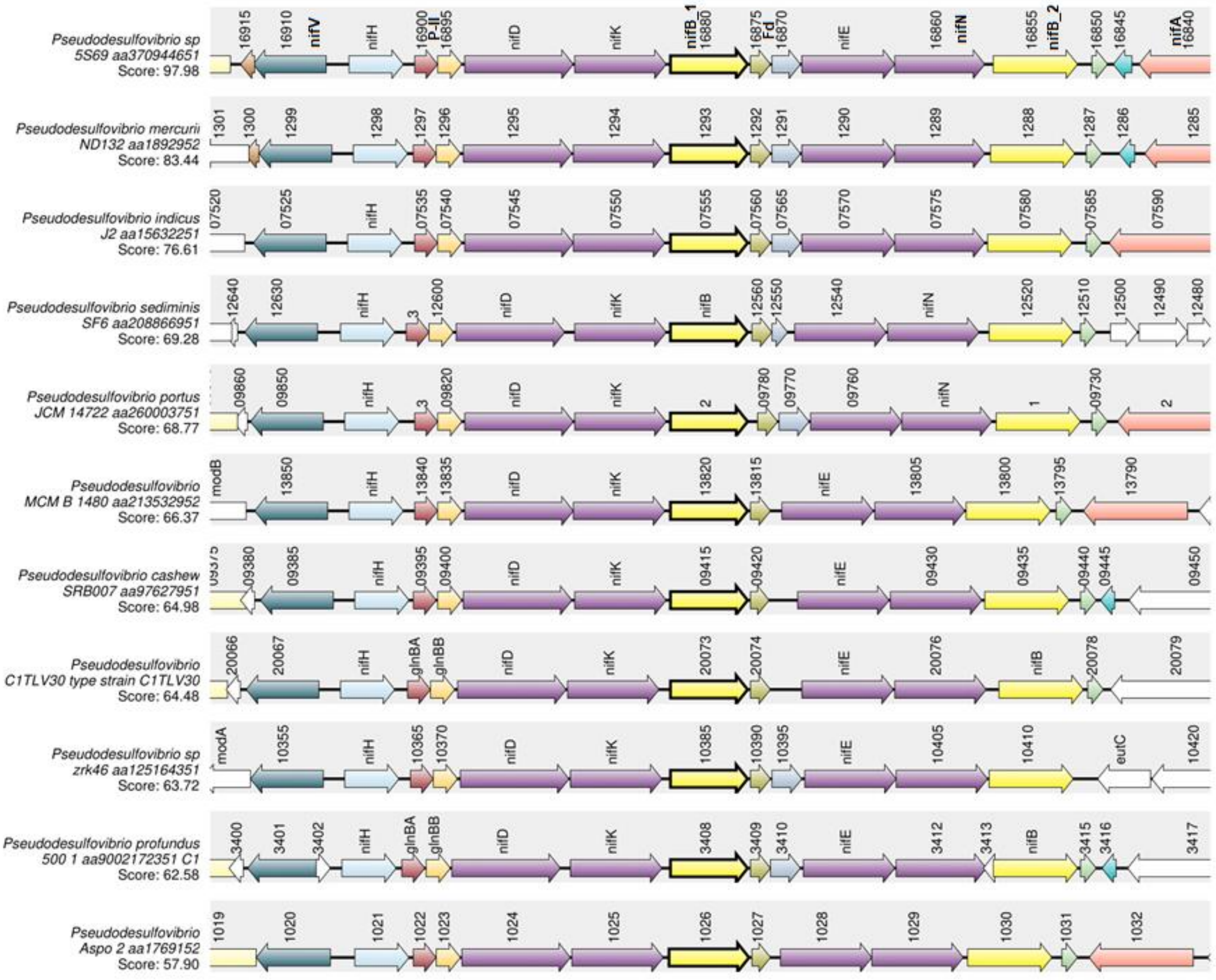

Figure S11. Organization of gene clusters presumably encoding nitrogen fixation enzymes in the genome of strain 5S69[T] and other type strains of *Pseudodesulfovibrio* species. Abbreviations: *nifH*, nitrogenase (molybdenum-iron) reductase and maturation protein; *nifDK*, nitrogenase (molybdenum-iron) alpha and beta subunits; *nifB_1-2*, nitrogenase FeMo-cofactor synthesis FeS core scaffold and assembly protein; Fd, ferredoxin; P-II, nitrogen regulatory proteins; *nifN*, nitrogenase FeMo-cofactor scaffold and assembly protein; *nifE*, nitrogenase iron-molybdenum cofactor biosynthesis protein; *nifA*, nitrogenase (molybdenum-iron)-specific transcriptional regulator; *nifV*, homocitrate synthase.

Table S1. Cellular fatty acid composition of strain 5S69^T and type strain of *Desulfovibrio desulfuricans* B-1799^T.

Fatty acid	Strain 5S69^T	Strain B-1799^T
iso-$C_{14:0}$	3.2	–
$C_{14:0}$	2.8	–
iso-$C_{15:0}$	**20.4**	**43.3**
anteiso-$C_{15:0}$	**19.3**	2.0
$C_{15:0}$	0.5	–
iso-$C_{16:1}$ ω9	2.5	2.1
iso-$C_{16:0}$	4.2	–
$C_{16:1}$ ω9	0.5	–
$C_{16:0}$	**16.3**	**7.1**
$C_{17:1}$ ω10c	2.9	–
anteiso-$C_{17:1}$ ω9	1.2	–
$C_{17:1}$ ω9c	3.8	**34.8**
$C_{17:1}$ ω9t	**6.6**	–
iso-$C_{17:0}$	3.5	**8.6**
anteiso-$C_{17:0}$	2.6	–
$C_{18:1}$ ω10	3.7	–
$C_{18:1}$ ω9c	1.8	–
$C_{18:1}$ ω9t	0.4	–
$C_{18:0}$	1.4	–
Others	2.4	2.1
Total	100.00	100.00

*The values are percentages (w/w) of total fatty acids. Dominant fatty acids are indicated in bold.
